# Risk factors for violence against women in high-prevalence settings: a mixed-methods systematic review and meta-synthesis

**DOI:** 10.1136/bmjgh-2021-007704

**Published:** 2022-03-16

**Authors:** Jenevieve Mannell, Hattie Lowe, Laura Brown, Reshmi Mukerji, Delan Devakumar, Lu Gram, Henrica A F M Jansen, Nicole Minckas, David Osrin, Audrey Prost, Geordan Shannon, Seema Vyas

**Affiliations:** 1 Institute for Global Health, UCL, London, UK; 2 Independent Researcher, Bangkok, Thailand; 3 Independent Researcher, London, UK

**Keywords:** public health, systematic review, epidemiology

## Abstract

**Introduction:**

Violence against women (VAW) affects one in three women globally. In some countries, women are at much higher risk. We examined risk factors for VAW in countries with the highest 12-month prevalence estimates of intimate partner violence (IPV) to develop understanding of this increased risk.

**Methods:**

For this systematic review, we searched PUBMED, CINAHL, PROQUEST (Middle East and North Africa; Latin America and Iberia; East and South Asia), Web of Science, EMBASE and PsycINFO (Ovid) for records published between 1 January 2000 and 1 January 2021 in English, French and Spanish. Included records used quantitative, qualitative, or mixed-methods, reported original data, had VAW as the main outcome, and focused on at least one of 23 countries in the highest quintile of prevalence figures for women’s self-reported experiences of physical and/or sexual violence in the past 12 months. We used critical interpretive synthesis to develop a conceptual model for associations between identified risk factors and VAW.

**Results:**

Our search identified 12 044 records, of which 241 were included for analysis (2 80 360 women, 40 276 men, 274 key informants). Most studies were from Bangladesh (74), Uganda (72) and Tanzania (43). Several quantitative studies explored community-level/region-level socioeconomic status and education as risk factors, but associations with VAW were mixed. Although fewer in number and representing just one country, studies reported more consistent effects for community-level childhood exposure to violence and urban residence. Theoretical explanations for a country’s high prevalence point to the importance of exposure to other forms of violence (armed conflict, witnessing parental violence, child abuse) and patriarchal social norms.

**Conclusion:**

Available evidence suggests that heightened prevalence of VAW is not attributable to a single risk factor. Multilayered and area-level risk analyses are needed to ensure funding is appropriately targeted for countries where VAW is most pervasive.

**PROSPERO registration number:**

The review is registered with PROSPERO (CRD42020190147).

**Key messages**

**What is already known?**

- There are large differences in the prevalence of violence against women both within and between countries.
- There is limited understanding of which contextual factors drive high rates of violence against women (VAW) in certain countries.
- Countries with the highest VAW prevalence estimates have largely been excluded from previous reviews.

**What are the new findings?**

- This is the first review of VAW risk factors to focus specifically on high-prevalence settings.
- Multiple, overlapping risks are responsible for a heightened prevalence of VAW, rather than a single factor.
- Population-level exposure to other forms of violence (armed conflict, witnessing parental violence, child abuse) and patriarchal social norms appear to drive high levels of violence against women.

**What do the new findings imply?**

- There is a dearth of evidence on how the risk factors operating at community, regional, national and global levels impact on violence, and on how risk factors may change over time.
- Additional longitudinal and cross-national analyses are needed to inform VAW interventions in high-prevalence settings.

## Introduction

Violence against women (VAW) has severe consequences for women’s health and well-being globally.^1^ While violence affects women in every country, it does so unevenly, with large differences in prevalence both within and between countries. Recent estimates suggest that between 10% and 53% of ever-partnered women have experienced physical and/or sexual violence by an intimate partner in their lifetime, with past 12-month prevalence estimates ranging between 2% and 36%.^2^ Some of the highest VAW prevalence estimates are found in informal settlements,^3^ Indigenous communities,^4^ conflict zones^5^ and certain regions of the world, such as the Pacific.^6^ Contextual factors proposed to shape violent behaviours towards women include inequalities in income and education, gendered cultural norms and practices, exposure to other forms of violence, and racial or class-based discrimination.^7 8^

Currently, however, there is limited understanding of which contextual factors drive high rates of VAW in certain settings. Armed conflict has been proposed as one such risk factor, as highlighted in Afghanistan and the Democratic Republic of Congo (DRC), where prevalence estimates of intimate partner violence (IPV) in the past 12 months measured 46% and 37%, respectively.^9 10^ Yet many countries with a high prevalence of IPV have not experienced recent conflict, including Fiji and the Marshall Islands (where lifetime exposure to IPV measured 64% and 51%, respectively).^11^ Feminist scholars have focused on patriarchal norms as a critical driver of VAW globally.^12^ However, countries such as Sweden and Denmark, where gender equality is supported by relevant policy and frameworks, have relatively high levels of lifetime exposure to physical and/or sexual violence (28% and 32% respectively), an anomaly often referred to as the Nordic paradox.^13^

Countries with the highest prevalence estimates have largely been excluded from previous reviews. Research from such countries often fails to meet methodological or review inclusion criteria,^14–17^ and recent reviews have tended to focus on risk factors rather than settings, including reviews of the public justification of violence,^18^ community-level correlates,^19^ child abuse,^20^ natural disasters,^21^ forms of violence^22^ or subpopulations such as pregnant women,^23 24^ and elderly women.^25^

To our knowledge, this is the first review of risk factors to focus on high-prevalence settings. It builds on other reviews that have taken an area or regional focus.^26–28^ We aim to identify the risk factors for VAW in the highest prevalence countries to (1) inform analyses of relationships between risk factors, (2) identify gaps to be addressed through further research and (3) inform policy priorities for the leave-no-woman-behind agenda.^29^ The review was designed to identify the broadest possible list of potential risk factors and draws together both quantitative and qualitative evidence.

## Methods

### Search strategy and selection criteria

We developed a rigorous search strategy (online supplemental file 1) and searched 19 databases for records in English, French or Spanish published between January 2000 and January 2021. The year 2000 represents the start of data collection using the WHO methodology, widely recognised as best practice for measuring VAW at a population level.^30^ Bibliographic databases included EMBASE, MEDLINE (PubMed), PsycINFO, Web of Science, CINAHL, Latin America & Iberia Database (ProQuest), Middle East & Africa Database (ProQuest), East and South Asia (ProQuest), Scielo, Latin America and Caribbean Health Science Library (PAHO). Additionally, we searched 10 databases for grey literature, including World Bank Open Knowledge Repository, WHO Prevent Violence Evidence Base and Resources, WHO Institutional Repository for Information Sharing, UNFPA regional websites, UNDP, UN Women, WHO Reproductive Health Library, Human Rights Watch, Relief Web, Observatorio de Igualdad de Género de América Latina y el Caribe. Search terms were divided into two strings: the first related to types of VAW, high-prevalence countries and risk factors; the second related to VAW in general, risk factor analyses and the global/cross-national level. We also identified records through expert referrals, handsearching relevant journals and citation chaining.

10.1136/bmjgh-2021-007704.supp1 Supplementary data

Twenty-three countries were identified as high-prevalence settings for the review. These countries represent the top quintile of prevalence figures for women’s self-reported experiences of physical or sexual violence in the past 12 months (table 1). By classifying high-prevalence countries according to currently available WHO data, we have only included countries for which these data were available.^31^

**Table 1:** Countries included in the review, by relevant characteristics

Country	Prevalence of past 12-month experience of physical and or sexual IPV (%)*	WHO region	GINI coefficient†	High/ middle/low income‡	Armed conflict since 1990§
Angola	25.9 (DHS 2016)	African	0.513	Lower-middle	Yes
Burundi	27.8 (DHS 2017)	African	0.386	Low	Yes
Cameroon	32.7 (MICS 2014)	African	0.466	Lower-middle	Yes
Central African Republic	26.3 (MICS 2006)	African	0.562	Low	Yes
DRC	36.8 (DHS 2014]	African	0.421	Low	Yes
Equatorial Guinea	43.6 (DHS 2011)	African	Not available	Upper middle	Yes
Gabon	31.5 (DHS 2012)	African	0.380	Upper middle	Yes
Liberia	36.3 (DHS 2007)	African	0.353	Low	Yes
Sierra Leone	28.7 (DHS 2013)	African	0.357	Low	Yes
Sao Tome and Principe	27.9 (DHS 2009)	African	0.563	Lower middle	No
Tanzania	29.6 (DHS 2016)	African	0.405	Lower middle	Yes
Uganda	29.9 (DHS 2016)	African	0.428	Low	Yes
Zambia	26.7 (DHS 2014)	African	0.571	Lower middle	No
Afghanistan	46.1(DHS 2015)	Eastern Mediterranean	Not available	Low	Yes
Bangladesh	28.8 (UNFPA 2015)	South-East Asian	0.324	Lower middle	Yes
Timor-Leste	34.6 (DHS 2016)	South-East Asian	0.287	Lower middle	Yes
Bolivia	27.1 (PAHO 2016)	The Americas	0.416	Lower middle	Yes
Fiji	29.7(National Research on Women’s Health and Life Experiences 2011)	Western Pacific	0.367	Upper middle	Yes
Kiribati	36.1(Family Health and Safety Study 2008)	Western Pacific	0.370	Lower middle	Yes
Micronesia	26.0 (Family Health and Safety Study 2014)	Western Pacific	0.401	Lower middle	No
Solomon Islands	41.8(Family health and safety study 2008)	Western Pacific	0.371	Lower middle	No
Tuvalu	25.0 (DHS 2007)	Western Pacific	0.391	Upper middle	No
Vanuatu	44.0 (National Survey on Women’s Lives and Family Relationships 2009)	Western Pacific	0.376	Lower middle	No

*Data compiled by the WHO as part of commitment to United Nations Sustainable Development Goals Intimate Partner Violence data indicator 5.2.1, https://unstats.un.org/sdgs/unsdg

†The GINI coefficient, a statistical representation of income inequality within a country that ranges from 0 (perfect equality) to 1 (perfect inequality), https://data.worldbank.org/indicator/SI.POV.GINI

‡Income classifications source: https://datahelpdesk.worldbank.org/knowledgebase/articles/906519-world-bank-country-and-lending-groups

§Heidelberg Institute for International Conflict Research HIIK database https://hiik.de/data-and-maps/datasets/?lang=en

DHS, Demographic and Health Surveys; DRC, Democratic Republic of Congo; IPV, intimate partner violence; MICS, Multiple Indicator Cluster Surveys.

The selection criteria used for abstract screening included primary research studies that were either qualitative (eg, used in-depth interviews, focus group discussions, or observations) or quantitative (eg, used cohort, case–control, cross-sectional or experimental designs). Titles and abstracts were double screened in Endnote by two reviewers (RM and LB) for records in high-prevalence countries that included at least one risk factor for violence against adult women (18 years or older) as the outcome. We excluded opinion pieces, editorials, policy briefs, general reports that did not present new empirical data, and conference abstracts. Disagreements over whether a record should be included were resolved by a third reviewer (JM).

We updated the initial database search in January 2021, resulting in 22 additional records, followed by a second search using a modified strategy that incorporated a new list of risk factors, resulting in 24 additional records. The list of risk factors for the first search was created from coding risk factors and relevant definitions from existing reviews and compiling these into a working template.^32^ For the second search, we modified this a priori template to include new risk factors or changes in definitions based on new codes identified during initial screening (online supplemental material), as a means of ensuring all potentially relevant risk factors had been included. No new risk factors were identified after the second search.

### Data analysis

Four reviewers (HL, RM, LB and JM) completed full text review of a subsection of records and extracted study characteristics, effect estimates and a summary of findings from included articles using a piloted form. Any discrepancy or query about data extraction was discussed by the review team. We developed a tailored approach to quality assessment which involved using set criteria as part of a fatal flaw analysis across all study types, consistent with the critical interpretive synthesis (CIS) approach used for meta-synthesis of the data (online supplemental materials).^33^ This approach to quality assessment prioritises the conceptual relevance of included studies over the degree to which they meet particular methodological standards for minimising the risk of bias, and is particularly useful for mixed-methods reviews that aim to make a theoretical or conceptual contribution.^34^

**Figure 1:**
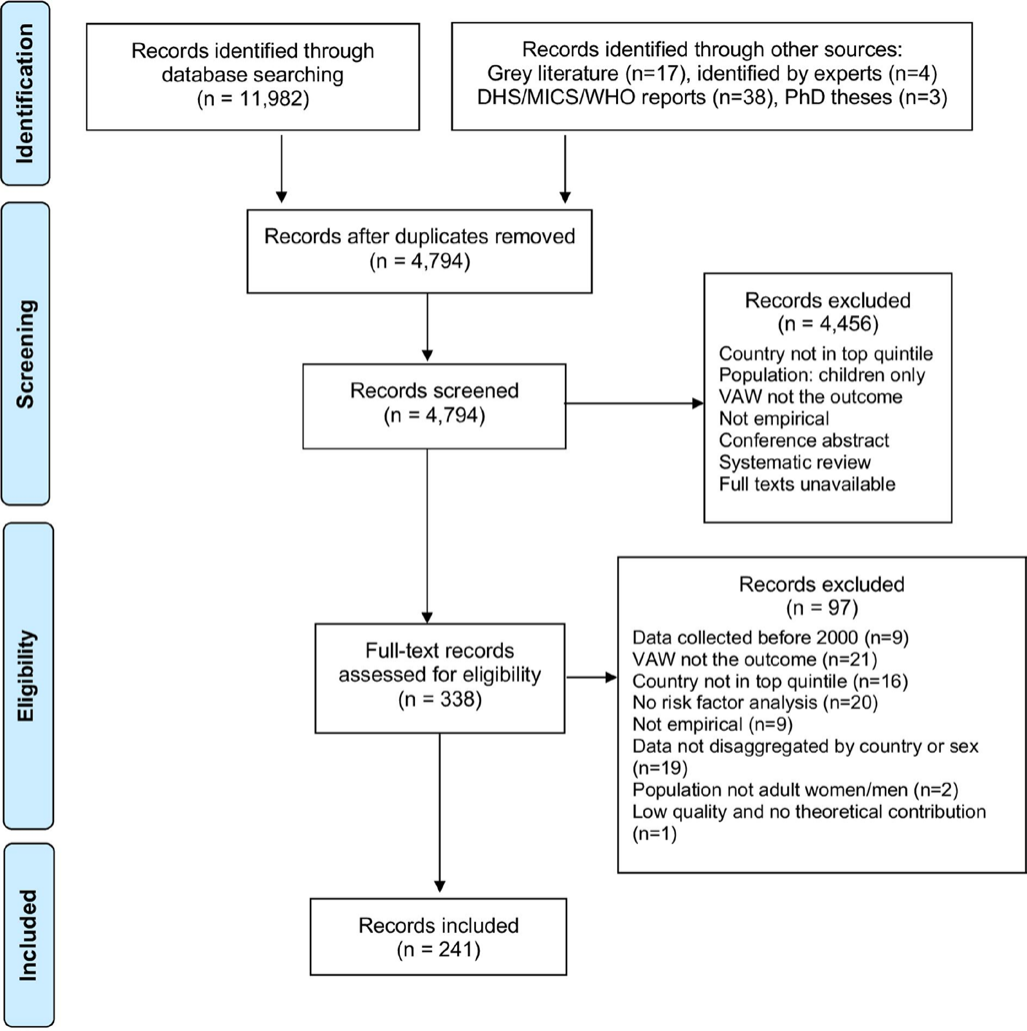
Study selection. Adapted from Moher *et al.*^189^ HS, Demographic and Health Surveys; MICS, Multiple Indicator Cluster Surveys; VAW, violence against women.

We first categorised identified risk factors thematically as part of a narrative literature summary. We then used CIS to generate theoretical insights from the integration of qualitative, quantitative and mixed-methods studies.^34^ CIS is differentiated from other meta-synthesis methods by its critical stance towards the presentation of the literature by primary authors, and its ability to generate theoretical insights through synthesis.^33^ HL and RM developed summary statements for each record (eg, how specific risk factors are linked to VAW), and grouped these statements into thematic categories. The review team then used these summary statements to develop a synthetic construct for how each risk factor was related to VAW and to other risk or protective factors. These synthetic constructs were linked together visually and formed our conceptual framework (figure 1).

We collated quantitative estimates measured at the community, district and region-level and presented these as ORs or incidence rate ratios in a forest plot to provide a visual summary of area-level risk factors, their effect sizes and directions of association with VAW. We presented area-level rather than individual-level estimates (and included studies that used aggregated individual data to create area-level measures), as these provide insights into contextual and structural factors shaping high prevalence settings. We decided against a meta-analysis because few records reported on the same risk factors at area level, and because of heterogeneity in how exposures and outcomes were measured.

## Results

**Figure 2:**
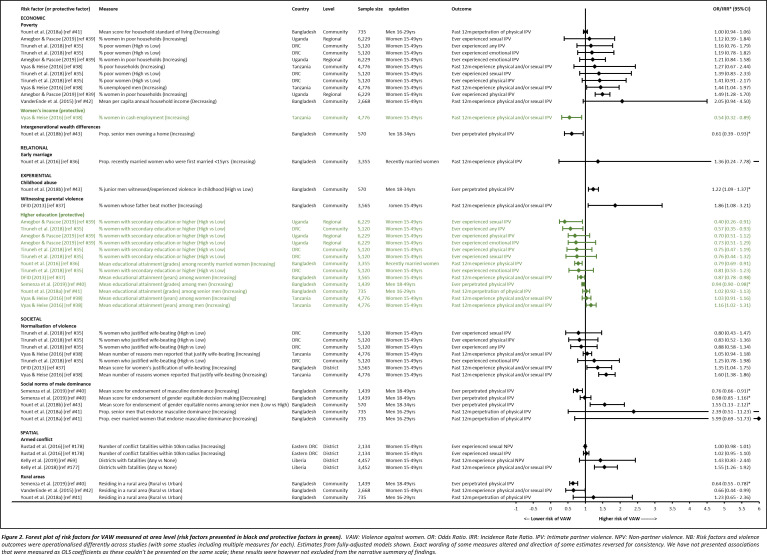
Forest plot of risk factors for VAW measured at area level. VAW, violence against women.

Our search identified 12 044 records. We screened titles and abstracts of 4794 unique records. A total of 338 met the criteria for full-text review. Records were excluded at the full review stage if VAW was not the outcome (n=21), there were no data on targeted countries (n=16), no risk factor analysis was included (n=20), data were not disaggregated by country or sex (n=19), no empirical data were provided (n=9), data were collected before 2000 (n=9), the population were not adult women or men (n=2), or the paper was of low quality and made no theoretical contribution (n=1) (see figure 2). Table 2 summarises characteristics of the 241 included records.

**Table 2:** Characteristics of included records

Characteristic	No of records (%)
Year of publication	
2000–2010	41 (17.0)
2011–2021	200 (83.0)
Publication type	
Peer-reviewed journal article	222 (92.1)
Grey literature report	12 (5.0)
DHS/MICS/WHO reports	4 (1.7)
PhD theses	3 (1.2)
Country	
Bangladesh	74
Uganda	72
Tanzania	43
Zambia	23
Democratic Republic of the Congo	23
Cameroon	12
Sierra Leone	10
Bolivia	10
Liberia	9
Timor-Leste	9
Afghanistan	8
Burundi	7
Gabon	6
Sao Tome and Principe	5
Angola	5
Central African Republic	2
Vanuatu	2
Micronesia	2
Kiribati	1
Solomon Islands	1
Fiji	1
Equatorial Guinea	0
Tuvalu	0
Data source	
Primary	133 (55.2)
Secondary	104 (43.2) (62% DHS)
Both primary and secondary	4 (1.6)
Methods	
Quantitative	175 (72.6)
Qualitative	58 (24.1)
Mixed	8 (3.3)
Study methods	
Quantitative designs	
Cross-sectional	169 (70.1)
Longitudinal (prospective cohort n=2, retrospective cohort n=1, longitudinal analysis of baseline/endline data n=2)	5 (2.1)
Retrospective	1 (0.4)
Qualitative methods	
Individual interviews	26 (10.8)
Focus group discussions	9 (3.7)
Ethnography	2 (0.8)
Case study	1 (0.4)
Combination of qualitative methods	20 (8.3)
Type of violence studied	
Physical	211
Sexual	163
Psychological	87
Economic	22
Controlling behaviour	11
Language	
English	239 (99.2)
Spanish	2 (0.8)
	Total studies=241

Percentages not included for country and type of violence because some studies included data from more than one country and for more than one type of violence.

**Figure 3:**
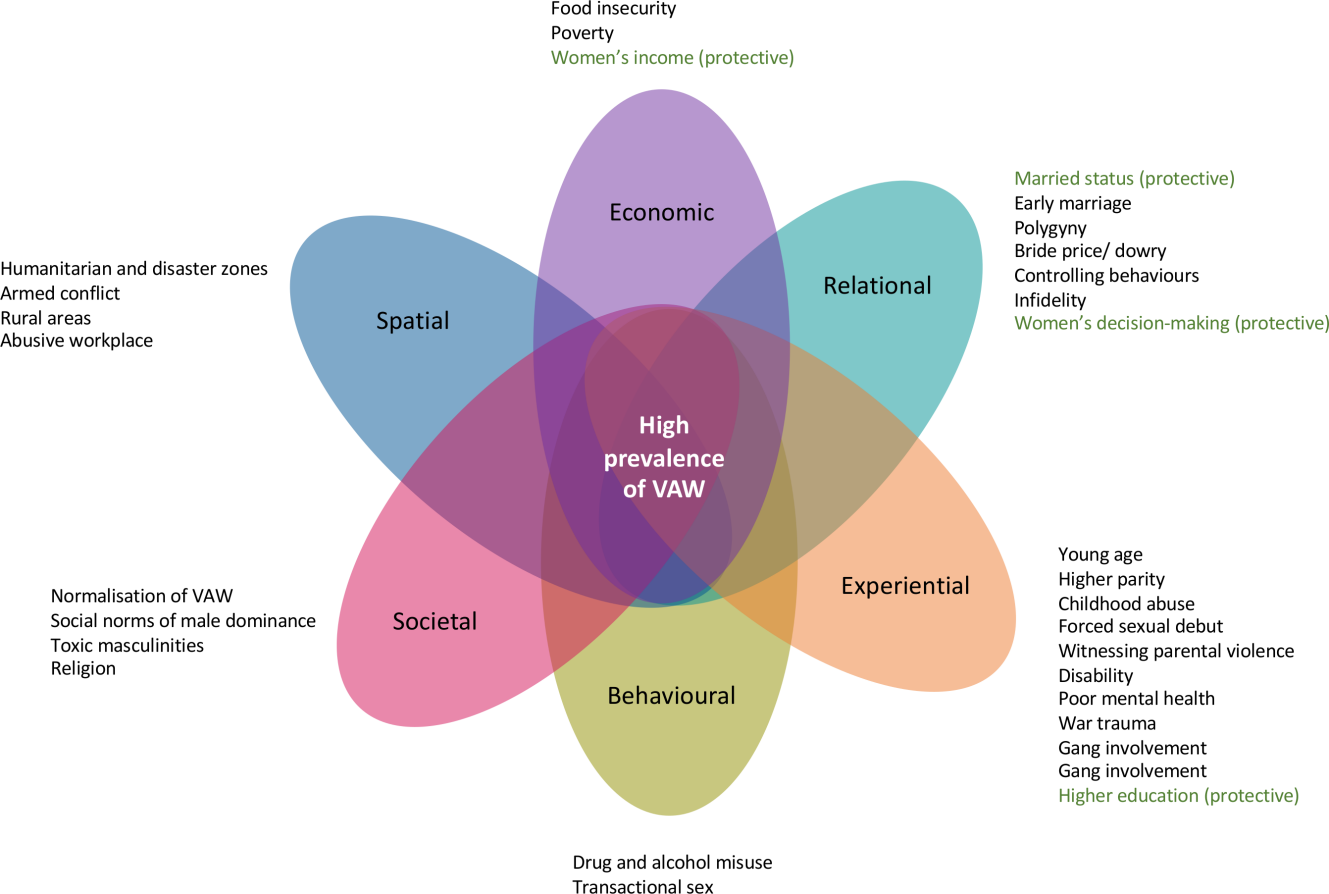
Overlapping risk factors for VAW in high-prevalence settings. VAW, violence against women.

DHS, Demographic and Health Surveys; MICS, Multiple Indicator Cluster Surveys.

figure 3 presents included quantitative studies that measured area-level (community, district, region) risk factors. This provides a visual account of the rather limited information currently available about associations between the characteristics of high-prevalence settings and VAW. Education was the most explored risk factor (with higher education seen as protective), with a total of seven separate studies from four different countries looking at its area-level association with VAW.^35–41^ However, with different directions of effect and not all associations significant, the evidence for the association between area-level education and VAW was mixed and appears to be context-specific.^35–41^ Area-level poverty was also relatively frequently explored, but while the five separate studies from four different countries that looked at this found consistent directions of association,^35 38 39 41 42^ only two were significant.^38 39^ Mixed directions of association were also found for normalisation of violence and social norms of male dominance, but the direction of the only significant associations^38 43^ suggested increasing normalisation was associated with increased risk of VAW. Community-level childhood abuse (men) and witnessing parental violence (women) were only looked at in one study each^37 43^ but statistically significant effect sizes suggest some evidence for their role in VAW perpetration and experience.

At the individual and relational level, the most commonly studied risk factors in quantitative analyses were education, age, alcohol use and socioeconomic status. Less commonly studied risk factors included natural and environmental disasters, male partner’s experiences of violence, social support networks (for non-pregnant women), and the influence of peer networks. A more substantial picture of the drivers of VAW in high-prevalence settings arises from the results of all quantitative, qualitative and mixed-methods studies together, which are organised thematically.

### Economic factors

Food insecurity was associated with different forms of VAW across numerous studies.^44–49^ An association between VAW and household socioeconomic status or asset wealth index was also observed at individual^50–54^ and regional levels.^55^ Theoretically, this association has been linked to how poverty-related stress increases the use of violence in households or between individuals who perceive it as an appropriate response to conflict.^56^ Contrary to theory, however, a study in Zambia found violence to be significantly higher among non-poor women compared with other women.^57^

Qualitative and quantitative data supported an association between women’s higher earnings and lower levels of past-year physical IPV.^58–60^ However, context played an important role. When women contributed equally or more than their husbands in Bangladesh, they were less likely to experience psychological, physical and sexual IPV than when their husbands contributed more or all income.^61^ In Tanzania, however, women had a greater risk of experiencing physical and sexual IPV when their financial contributions were greater than their partners’.^58^ In Bangladesh, young men were also less likely to perpetrate physical IPV in communities where more senior men owned homes, suggesting that intergenerational wealth differences may also be a risk factor for VAW.^43^

### Relational factors

Compared with being unmarried and being in a cohabiting relationship, being married offered women protection against IPV in several studies.^41 49–51^ This finding could align with commitment theories that assert that cohabitation is an indicator of weakened relationships,^62^ or it could be that women are less likely to marry their violent partners. Being married was also protective against IPV when compared with being separated or divorced,^63 64^ although this may also be a reflection of women who experience violence leaving their violent partners. However, being married was found to increase the risk of sexual IPV compared with being unmarried,^65 66^ while single and unmarried women appeared to be at the highest risk of non-partner sexual^67^ and physical violence.^68^

Younger age at marriage was associated with increased domestic violence (from a partner or other family member),^69^ and with IPV.^52 70–73^ An association between the village-level prevalence of child marriage (<15 years) and IPV was found in Bangladesh, suggesting that women who lived in villages with high levels of child marriage were also at increased risk of IPV even if they married as adults themselves.^36^ In Afghanistan, women who were married before age 15 were reported to have a higher risk of sexual violence, compared with those married as adults (≥18 years).^74^ Generally, the risk of past year IPV reduced as marital duration increased,^75–78^ while lifetime IPV increased.^72 79 80^

Polygyny, often classified as an indicator of gender inequality, was strongly associated with increased IPV across several studies.^44 81–84^ A qualitative study from Bangladesh suggested polygyny created conflict between partners, with several female participants believing their inquiries about co-wives led to them being physically beaten.^85^ In Uganda, unequal love, neglect and jealousy created conflict in relationships that led to IPV.^86^ In Tanzania, polygyny was an indicator of women’s lower status, which increased their vulnerability to IPV.^60^

Women whose marriages involved dowry payment were more likely to experience IPV than women whose marriages did not.^87–90^ This was supported by qualitative data from Uganda and Tanzania where bride price was perceived to worsen gender inequalities by representing women as ‘bought’, reducing their decision-making power and increasing their risk of violence.^91–93^ Issues surrounding unpaid or partially paid dowry were highlighted as an additional source of relationship conflict triggering violence.^85 94^

There were strong, consistent associations between controlling behaviours and multiple types of IPV (physical, sexual and psychological) across countries.^35 49 51 54 61 72 80 88 95–101^ A study in Uganda found that male partners’ controlling behaviours were the strongest predictors of sexual IPV.^102^ This was supported by qualitative evidence demonstrating how IPV ensures a woman’s submissiveness and obedience and reaffirms her partner’s perceived masculinity,^85 103^ consistent with theoretical assertions that coercive control forms part of the overall pattern of women’s experiences of violence.^104^

A number of studies demonstrated that women who were involved in more egalitarian household decision making were less likely to experience IPV.^35 59 70 105–107^ However, a study from Bangladesh found that women’s risk of experiencing physical or sexual IPV increased with greater participation in household decision making.^108^

Infidelity by men was found to be a risk factor for IPV in both quantitative and qualitative studies.^54 100 101 109–111^ In Bangladesh, women whose partners had other sexual relationships were more likely to experience IPV than women whose partners did not.^87^ Moreover, when men suspected their female partner’s infidelity, or vice versa, women were more likely to experience IPV.^51 60 98^

### Experiential factors

Young age was associated with increased risk of VAW in many studies.^72 81 106 111–119^ Ever-married or cohabiting women aged 20–24 years were significantly more likely to experience past year physical or sexual IPV than women aged 35 years or over.^117^ Having a greater number of children was also associated with an increased risk of IPV among women,^49 59 63 64 69 72 106 111 113 120–125^ possibly suggesting higher parity reinforces structural norms keeping women dependent on their partners.

Childhood abuse was associated with IPV perpetration (among men) or victimisation (among women) in a large number of studies.^48 53 54 96 100 101 111 116 126 127^ In Cameroon, a woman’s experience of physical abuse in childhood predicted sexual IPV victimisation in adulthood, which the authors suggested was the result of poor conflict resolution skills in adulthood.^128^ Childhood maltreatment was a strong predictor of IPV perpetration in adulthood among men in Burundi.^129^ Across many countries, forced sexual debut was a risk factor for IPV in later life among female sex workers,^88 126 130 131^ and among adolescent girls and young women.^111 116 132^

Witnessing IPV between parents during childhood was associated with IPV victimisation and perpetration in adulthood at the individual level,^50 53 54 72 82 101 111 133^ and at the community level.^37^ Qualitative data from Uganda highlighted negative role modelling in families and how boys from households with parental IPV were growing up to become violent husbands themselves.^12^

Women with disabilities were more likely to experience physical, sexual and emotional violence than women without disabilities.^134–137^ Qualitative findings from the DRC suggested that this may be related to an inability to fulfil expected gender roles in the household.^138^ Women who experienced mental ill health were similarly at risk of experiencing violence in intimate relationships and family settings,^46 47 63 139^ but also in the workplace^140^ and by strangers.^47 141^ VAW was perpetrated by caregivers, partners (including men who also suffered with poor mental health) and strangers.^96 142–144^

In conflict and postconflict settings including Afghanistan, Liberia, Uganda and Sierra Leone, war violence exposure was associated with increased IPV perpetration among men and IPV victimisation among women.^46 139 145–147^ In Liberia, this association was independently mediated by anxious attachment styles and attitudes justifying wife beating.^139^ In South Kivu, DRC, men discussed how their experiences of wartime violence were a risk factor for their perpetration of rape.^148^ In Timor Leste, women linked men’s war exposure to increased emotional problems and alcohol consumption, increasing IPV risk in the home.^149^ Men who were involved with gangs were more likely to perpetrate both physical and sexual IPV,^96^ as were men involved in fights with other men.^95 100 101 111 132^

Although the effect of education on VAW varied across different geographies and population groups, higher levels were generally associated with reduced violence perpetration and victimisation.^35 50 86 87 112 120^ Women’s higher education protected against IPV at both the individual and community level in Bangladesh and DRC,^35 37^ and at the regional level in Uganda.^39 150^ Participants in Bangladesh linked high rates of VAW to poverty and a lack of education, suggesting that deprivation leads to violence,^94^ while others suggested that women’s increased education reduced IPV through expanding opportunities for income generation.^151^

### Behavioural

Several studies across countries supported the hypothesis that alcohol use affects cognitive functioning, raises aggression and increases men’s perpetration of VAW.^35 48 50 54 82–84 100 101 111 152 153^ Partner’s illicit drug use was also associated with increased IPV perpetration in Vanuatu^154^ and Bangladesh.^75 120^

Past year transactional sex was associated with increased verbal, physical, and sexual IPV among women in Uganda.^155 156^ Adolescent girls and young women who were out of school and engaged in sex work within the past 6 months in Tanzania were also at increased risk of IPV.^63^ Evidence from Cameroon, the DRC and Uganda points to how stigmatisation and structural discrimination of sex work leads to further violence, for example, from the police.^130 157–159^

### Societal factors

A large number of studies found that acceptance of violence among both men and women was a strong predictor of men’s perpetration and women’s experiences of different types of violence,^50 54 68 77 79 80 83 87 100 101 116 126 133 139 160^ with the strongest associations found when both partners supported the use of violence in relationships.^82 161^ Similarly, the severity of IPV increased when male partners had more accepting attitudes to violence.^162^ This widespread normalisation of violence was evident in qualitative reports of misogyny in Afghanistan’s social and institutional frameworks,^163^ and quantitative evidence of an association between community-level attitudes that support the use of VAW in relationships and an increase in women’s experiences of IPV in Tanzania.^38^

There is strong qualitative evidence supporting the role of patriarchal social norms and masculine ideals in contributing to VAW. In Bangladesh, men’s views on gender and sexuality were aligned with patriarchal norms suggesting that wives should obey their husbands, which helped justify VAW when women transgressed or men felt the need to reinforce these gender roles.^12 164^ In Tanzania and postconflict DRC, men discussed using violence against their wives to reassert their authority and position as household head.^165 166^ Hegemonic masculinities that see a violent man as the ideal also contributed to IPV.^167^

Religious affiliation, often measured as a sociodemographic variable, was associated with IPV in several studies.^79 84 120 161 168–170^ However, this was context-specific: while two studies found that identifying as Muslim was protective against IPV,^120 161^ others showed an increased risk associated with identifying as Muslim,^84 89 169^ or belonging to ‘other religions’.^79 170^

### Spatial

Two studies explored violence against internally displaced persons (IDPs) in refugee camps. In an IDP camp in Northern Uganda, participants described how existing drivers of VAW, such as gender inequality, economic deprivation and alcohol abuse, were exacerbated and led to increased VAW, as did the physical layout and social characteristics of the camp itself.^171^ Qualitative data from four refugee camps in Uganda suggested that unequal power relations, poverty and unequal access to resources increased VAW.^172^

In Northern Uganda and Afghanistan, armed conflict has exacerbated existing structural factors that contribute to VAW, including gender inequalities, police corruption and poverty.^163 173 174^ Living in districts that experienced conflict increased women’s risk of experiencing IPV and non-partner sexual violence at the district level.^68 175 176^ Rape and gang rape of civilian women was widespread in conflict settings, with violent events often taking place in women’s homes during the night.^67 177^

Two studies explored the relationship between environmental shocks and VAW.^178 179^ In a qualitative study in Bangladesh, participants perceived VAW as having worsened immediately before, during, and after cyclones due to the need to move to shelters, staying in damaged homes and having to travel to collect relief and receive healthcare.^179^

Women living in rural areas were at greater risk of IPV than women in urban areas in Afghanistan, Liberia, Zambia and Bangladesh.^74 81 86 161 180^ Conversely, living in a rural area protected women against IPV in Zambia, Bolivia and Tanzania.^53 79 113^ One study suggested that urban social environments may be more stressful triggering conflict in relationships that leads to violence.^79^

**Figure 4:**
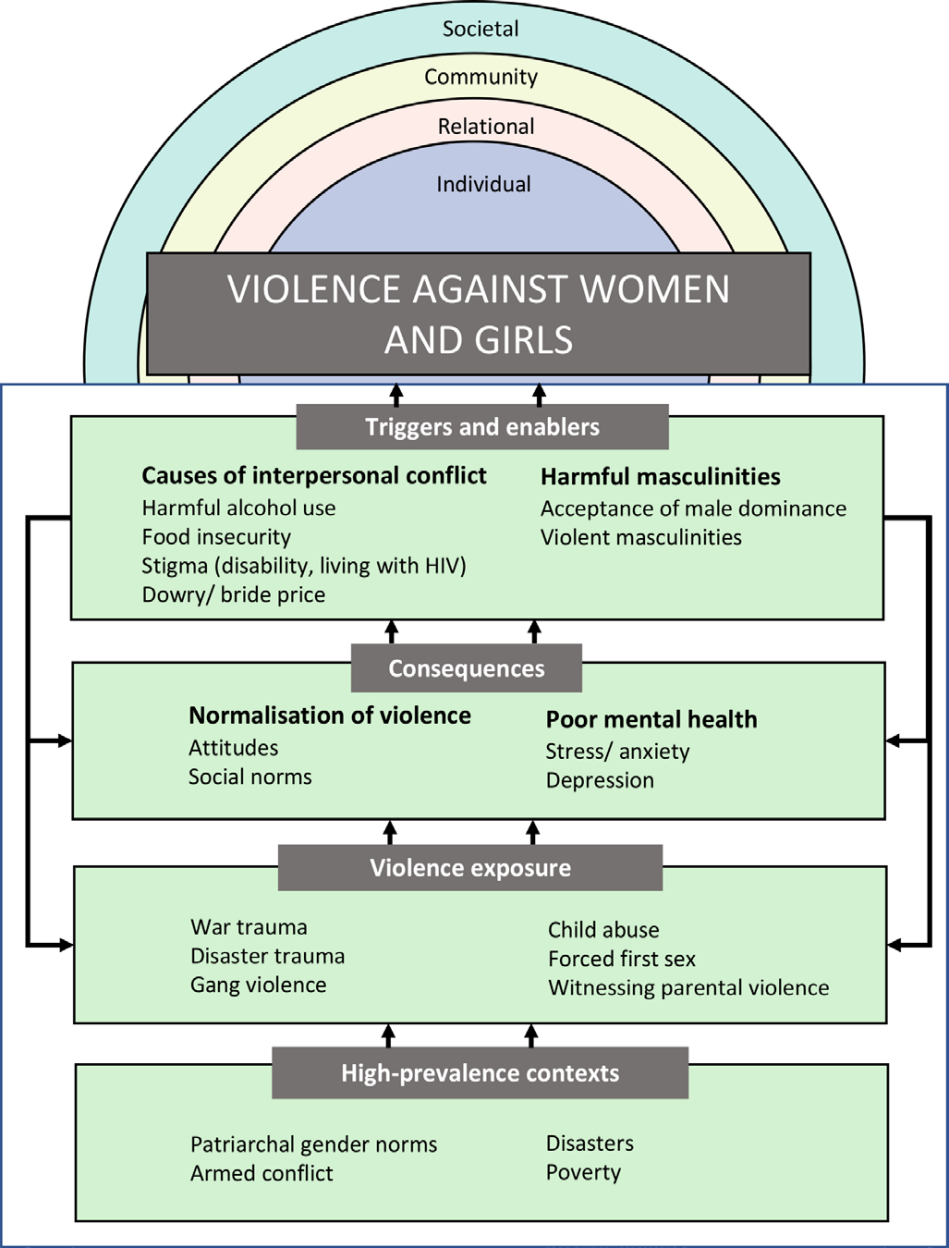
Conceptualising pathways of how structural country characteristics contribute to high VAW prevalence. VAW, violence against women.

A study in Bangladesh examined violence experienced by women working in the garment industry, including emotional abuse, physical and sexual violence, and economic control in the workplace and the home.^181^ Another study, also from Bangladesh, suggested that workplace VAW was driven by manager pressure to ensure intense productivity and the hierarchical structure within factories that fostered a culture of violence.^140^

figure 4 visually represents risk factors identified in all quantitative, qualitative and mixed-methods records. It highlights how overlapping categories magnify the risk of experiencing violence for women living in high-prevalence settings.

For example, VAW may result from the widespread social acceptance of violence, economic challenges that magnify interpersonal conflict, gender norms that condone male violence towards women, or as a response to unresolved trauma. Studies from high-prevalence countries included in this review draw attention to the extent of the violence in contexts where there is evidence all of these factors occur at the same time.

## Discussion

This is the first review, to our knowledge, to assess risk factors for VAW with a focus on high-prevalence countries. The evidence suggests that multiple and overlapping risk factors drive high rates of VAW in these settings, rather than a single risk factor such as armed conflict or gender inequalities. While some risk factors can be considered ‘universal’ with robust support across countries at both individual and area levels (ie, child marriage, child abuse, witnessing parental IPV, social norms of violence and hegemonic masculinities of men as naturally violent), other risk factors behave differently in different contexts, including education, women’s employment and religious affiliation.

These findings point to several potential pathways between risk factors and VAW. The theorised pathways with the strongest supporting evidence across settings are summarised in figure 1. Structural characteristics observed in many high-prevalence settings (eg, armed conflict, gender inequality, widespread poverty) expose large numbers of people to violent events, increasing mental ill health and consolidating acceptance of violence as normal. This subsequently instigates VAW when interpersonal relationships are arranged patriarchally and interpersonal conflict is triggered (eg, via harmful alcohol use, food insecurity, stigma). This conceptualisation of how area-level risks lead to the use of violence within interpersonal relationships contributes to recent discussions of how risk factors for VAW may be interrelated.^7^

Our review highlights notable gaps in analyses of risk factors at an area level (including regional, national and global spheres). Global drivers of risk for VAW are increasingly recognised as important,^182^ but vastly understudied in high-prevalence settings. This obscures critical understandings of how financial flows, remittances and global aid might influence the national prevalence of VAW,^183^ or the role of global communication and new technology in the rise of alternative forms of violence, including cyber sexual abuse and trafficking.^184^ Other sizeable gaps include studies of forms of violence other than IPV and non-partner sexual violence. Although IPV is the most common form of VAW globally,^1^ the focus on IPV has largely obscured attention to VAW in obstetrics,^185^ child and forced marriage^186^ and femicide.^187^

Perhaps most surprising is that after over 20 years of VAW risk factor research and thousands of published studies on the topic, our capacity to draw meaningful conclusions about why some countries have higher rates of VAW than others is limited. Many countries with the highest prevalence of VAW are under-researched for example, Fiji, Equatorial Guinea and Tuvalu. Most research in high-prevalence settings focuses on only three countries: Bangladesh, Uganda and Tanzania (67% of included studies). There are also few longitudinal or cross-national risk factor studies, leaving a weak body of evidence around the context-specific nature of risks, which risk factors are potentially modifiable or how they may change over time.

Inevitably, the recent COVID-19 pandemic has also changed patterns of risk for VAW in high-prevalence settings. There has been an increase in evidence around the impact of lockdown measures on the perpetuation of VAW globally,^188^ which has not been captured by this review. While the evidence synthesised in the review remains relevant for thinking about contextual risks more broadly, the pandemic is likely to corroborate evidence on the role of natural disasters in perpetrating VAW.

The review has several limitations. First, we decided against a broader review strategy that would allow for a comparison between risk factors in high-prevalence settings with risk factors in countries with lower prevalence. We decided to prioritise the identification of risk factors from both qualitative and quantitative evidence from an under-represented list of countries over and above this comparative analysis. As highlighted by our results, there is a need for more cross-comparative analyses of VAW risk and its associations with national characteristics, but these are better suited to quantitative analyses of secondary data than systematic review methods. The measure used to assess high prevalence in this review was past year physical and/or sexual IPV because of widespread availability of these data as part of Sustainable Development Goal country assessments, but this may have limited the inclusion of countries that experience high levels of other forms of VAW, notably Papua New Guinea. The limited number of studies examining area-level risk factor for VAW constrain what we were able to say about high-prevalence settings, and we were unable to compare differences in prevalence and associated risk factors within countries, which would be useful areas for future research. In addition, some countries included in the review may have publication records in languages other than English, French or Spanish (eg, Angola) that were not found through our search.

While an extensive body of evidence exists on risk factors for VAW globally, the breadth of research is limited for the highest prevalence countries. Extensive researcher time and energy have gone into secondary analyses of Demographic and Health Surveys data. This has been helpful in mapping context-specific risk profiles, but VAW research in high-prevalence settings must expand beyond a handful of well-researched countries and risks. Further area-level analyses that look at under-researched contexts, forms of violence and protective factors are needed to inform future interventions in areas where VAW is pervasive.

Addressing multiple intersecting forms of violence and discrimination as part of the leave-no-woman-behind agenda will require improved understandings of how certain contexts can contribute to a woman’s increased risk of violence. At a global level, such analyses can help contribute to more targeted and appropriate multicomponent programming for VAW prevention programmes, offering a valuable tool for international donors. For policy-makers in countries with high VAW prevalence, better understandings of the contextual factors driving within-country variations are essential for addressing structural inequalities and uneven access to existing services, and for identifying protective factors that could be better leveraged as part of national strategies. However, this requires a meaningful shift away from national analyses of risk factors and towards more advanced understandings of the contexts that create them.

## Data Availability

All data relevant to the study are included in the article or uploaded as online supplemental information. Not applicable.
