# Supplementary material for: Risk factors for violence against women in high-prevalence settings: a mixed-methods systematic review and meta-synthesis
Source: BMJ Glob Health. 2022 Mar 16;7(3):e007704. doi: 10.1136/bmjgh-2021-007704 (PMC8928330; doi:10.1136/bmjgh-2021-007704)
Supplement: Supplementary data [file bmjgh-2021-007704supp001.pdf]

Supplementary material:
Risk factors for violence against women in high-prevalence settings: A mixed methods systematic review and meta-synthesis

Contents

Table 1: Search strategy for risk factors of VAW in the highest prevalence settings
Table 2: Search strings
Table 3: Inclusion criteria
Table 4: Risk factor template
Table 5: Criteria for assessing fatal flaws in study design
Table 6: All included records, by study
Table 7: Quality assessment

Mannell J, *et al. BMJ Global Health* 2022; 7:e007704. doi: 10.1136/bmjgh-2021-007704

Supplementary material:
Risk factors for violence against women in high-prevalence settings: A mixed methods systematic review and meta-synthesis

Table 1: Search strategy for risk factors of VAW in the highest prevalence settings: [#2 AND #3 AND #4] + [#1 AND #2 AND #4 AND #5]

	Concept 1	Concept 2	Concept 3	Concept 4	Concept 5
**Key concepts**	**(1) Violence against women**	**(2) Types of violence**	**(3) High-prevalence settings**	**(4) Risk analysis**	**(5) Global**
**Free text terms / natural language terms**<br>(synonyms, UK/US terminology, medical/laymen's terms, acronyms/abbreviations, drug brands, more narrow search terms)<br>*Consider: phrase searching, proximity operators, truncation, wildcards, field qualification (e.g. textword)*	"violence against women"<br>"gender-based violence"<br>"gender violence"<br>"gender-associated violence"<br>"gender-related violence"<br>"women's health"<br>"women's rights"	"intimate partner violence" [MeSH]<br>"domestic violence" [MeSH]<br>"spouse abuse" [MeSH]<br>"battered women" [MeSH]<br>"family violence"<br>"partner abuse"<br>maltreatment<br>"marital rape"<br>"forced sex"<br>"non-partner sexual violence"<br>killings<br>"forced marriage"	Equatorial Guinea<br>Afghanistan<br>Vanuatu<br>Solomon Islands<br>Kiribati<br>Democratic Republic of the Congo<br>Liberia<br>Timor-Leste<br>Gabon<br>Cameroon<br>Fiji<br>Uganda<br>Tanzania<br>Burundi<br>Sierra Leone<br>Bangladesh<br>Bolivia<br>Micronesia<br>Zambia<br>Sao Tome and Principe<br>Central African Republic<br>Tuvalu<br>Angola	"risk factor" [MeSH]<br>"socioeconomic factors" [MeSH]<br>"social determinants of health" [MeSH]<br>"risk marker"<br>predictor<br>pathway<br>correlate*<br>driver<br><br>Specific risk factors:<br>education<br>age<br>alcohol<br>"social norm"<br>attitude<br>"substance use"<br>"employment status"<br>"child abuse"<br>ethnicity<br>married<br>religion<br>"male authority"<br>disability<br>"mental health"<br>conflict<br>poverty	global<br>multinational<br>multi country<br>"cross national"<br>international

Mannell J, *et al. BMJ Global Health* 2022; 7:e007704. doi: 10.1136/bmjgh-2021-007704

Supplementary material:
Risk factors for violence against women in high-prevalence settings: A mixed methods systematic review and meta-synthesis

	Concept 1	Concept 2	Concept 3	Concept 4	Concept 5
				“gender inequality”<br><br>Risk factors added after first round search<br>“microcredit participation”<br>“HIV status”<br>“witnessing violence”<br>“natural disaster”<br>“food insecurity”<br>parity<br>“sex work”<br>infidelity<br>polygamy<br>“duration of marriage”<br>income<br>dowry<br>autonomy<br>empowerment<br>“circumstances of first sex”<br>“media exposure”<br>“peer network”	

Supplementary material:
Risk factors for violence against women in high-prevalence settings: A mixed methods systematic review and meta-synthesis

Table 2: Search strings

**Database**	**Search string** (second round search highlighted)	**Results (#)**
PUBMED/ CINAHL	(1) ((((((((((((\"domestic violence\"[MeSH Terms]) OR (\"intimate partner violence\"[MeSH Terms])) OR (\"spouse abuse\"[MeSH Terms])) OR (\"battered women\"[MeSH Terms])) OR (family violence)) OR (partner abuse)) OR (maltreatment)) OR (marital rape)) OR (forced sex)) OR (non-partner sexual violence)) OR (honour killings)) OR (forced marriage) AND (2000:2021[pdat])) AND ((((((((((((((((((((((((equatorial guinea) or (afghanistan)) or (vanuatu)) or (solomon islands)) or (kiribati)) or (democratic republic of the congo)) or (liberia)) or (timor-leste)) or (gabon)) or (cameroon)) or (fiji)) or (uganda)) or (tanzania)) or (burundi)) or (sierra leone)) or (bangladesh)) or (bolivia)) or (micronesia)) or (zambia)) or (sao tome and principe)) or (central african republic)) or (tuvalu)) or (angola)) AND (2000:2021[pdat]))) AND ((\"socioeconomic factors\"[MeSH Terms]) OR (\"risk factors\"[MeSH Terms])) OR (\"social determinants of health\"[MeSH Terms])) OR (risk marker)) OR (predictor)) OR (pathway)) OR (correlate*)) OR (driver)) OR (education)) OR (age)) OR (alcohol)) OR (social norm)) OR (attitude)) OR (substance use)) OR (employment status)) OR (child abuse)) OR (jealousy)) OR (ethnicity)) OR (married)) OR (religion)) OR (male authority)) OR (poverty)) OR (disability)) OR (mental health)) OR (gender inequality)) OR (armed conflict) OR (microcredit participation)) OR (HIV status)) OR (witnessing violence)) OR (natural disaster)) OR (food insecurity)) OR (parity)) OR (sex work)) OR (infidelity)) OR (polygamy)) OR (duration of marriage)) OR (income)) OR (dowry)) OR (autonomy) OR (empowerment)) OR (circumstances of first sex)) OR (media exposure)) OR (peer network)) AND (2000:2021[pdat]))<br>(2) (((((\"violence against women\"[All Fields]) OR (\"gender based violence\"[All Fields])) OR (\"gender violence\"[All Fields]) AND (2000:2021[pdat])) AND (((((\"global\"[All Fields]) OR (multinational)) OR (\"cross national\"[All Fields])) OR (\"multi country\"[All Fields])) OR (international) AND (2000:2021[pdat]))) AND (((\"socioeconomic factors\"[MeSH Terms]) OR (\"risk factors\"[MeSH Terms])) OR (\"social determinants of health\"[MeSH Terms])) OR (risk marker)) OR (predictor)) OR (pathway)) OR (correlate*)) OR (driver)) OR (education)) OR (age)) OR (alcohol)) OR (social norm)) OR (attitude)) OR (substance use)) OR (employment status)) OR (child abuse)) OR (ethnicity)) OR (married)) OR (religion)) OR (male authority)) OR (poverty)) OR (disability)) OR (mental health)) OR (gender inequality)) OR (armed conflict)) OR (microcredit participation)) OR (HIV status)) OR (witnessing violence)) OR (natural disaster)) OR (food insecurity)) OR (parity)) OR (sex work)) OR (infidelity)) OR (polygamy)) OR (duration of marriage)) OR (income)) OR (dowry)) OR (autonomy) OR (empowerment)) OR (circumstances of first sex)) OR (media exposure)) OR (peer network)) AND (2000:2021[pdat]))) AND ((((((((((((\"domestic violence\"[MeSH Terms]) OR (\"intimate partner violence\"[MeSH Terms])) OR (\"spouse abuse\"[MeSH Terms])) OR (\"battered women\"[MeSH Terms])) OR (family violence)) OR (partner abuse)) OR (maltreatment)) OR (marital rape)) OR (forced sex)) OR (non-partner sexual violence)) OR (honour killings)) OR (forced marriage) AND (2000:2021[pdat]))	PUBMED results = (1)997 + (2)555 = 1,552 [Search date 05/01/21]<br><br>CINAHL results = (1)301 + (2)0 = 301 [Search date 05/01/21]

Supplementary material:
Risk factors for violence against women in high-prevalence settings: A mixed methods systematic review and meta-synthesis

**Database**	**Search string** (second round search highlighted)	**Results (#)**
PROQUEST Middle East and North Africa/ Latin America and Iberia/ East and South Asia	(1) "violence against women" or "gender-based violence" or "gender violence"<br>(2) "intimate partner violence" or "domestic violence" or "family violence" or "partner abuse" or "spous* abuse" or "battered women" or maltreatment or "marital rape" or "forced sex" or "non-partner sexual violence" or "hono* killings" or "forced marriage"<br>(3) "equatorial guinea" or afghanistan or vanuatu or "solomon islands" or Kiribati or "democratic republic of the congo" or Liberia or timor-leste or gabon or Cameroon or fiji or Uganda or tanzania or burundi or "sierra leone" or bangladesh or bolivia or micronesia or zambia/ or "sao tome" or "central african republic" or tuvalu or angola<br>(4) "risk marker" or "risk factor" or predictor or correlate or driver or "socioeconomic factors" or "social determinants" or age or alcohol or "social norm" or attitude or employment or "child abuse" or married or religion or "male authority" or disability or "mental health" or conflict or poverty or "gender inequality" or "microcredit participation" or "HIV status" or "witnessing violence" or "natural disaster" or "food insecurity" or "parity" or "sex work" or "infidelity" or "polygamy" or "duration of marriage" or "income" or "dowry" or "autonomy" or "empowerment" or "circumstances of first sex" or "media exposure" or "peer network"<br>(5) global or multinational or "multi country" or "cross national" or international	MIDDLE EAST NORTH AFRICA results = (2,3,4)456 + (1,2,4,5)461 = 917 [Search date 05/01/21]<br><br>EAST AND SOUTH ASIA results = (2,3,4)121 + (1,2,4,5)139 = 260 [Search date 06/01/21]<br><br>LATIN AMERICA AND IBERIA results = (2,3,4)161 + (1,3,4,5)386 = 547 [Search date 05/01/21]
WEB OF SCIENCE	(1) ("intimate partner violence" OR "domestic violence" OR "family violence" OR "partner abuse" OR "spous* abuse" OR "battered women" OR maltreatment OR "marital rape" OR "forced sex" OR "non-partner sexual violence" OR "hono* killings" OR "forced marriage") AND ("equatorial guinea" OR afghanistan OR vanuatu OR "solomon islands" OR kiribati OR "democratic republic of the congo" OR liberia OR timor-leste OR gabon OR cameroon OR fiji OR uganda OR tanzania OR burundi OR "sierra leone" OR bangladesh OR bolivia OR micronesia OR zambia OR "sao tome" OR "central african republic" OR tuvalu OR angola OR kenya) AND ("risk marker" OR "risk factor" OR predictor OR correlate OR driver OR "socioeconomic factors" OR "social determinants" OR age OR alcohol OR "social norm" OR attitude OR employment OR "child	Web of Science results = (1)1,247 + (2)1,035 = 2,282

Supplementary material:
Risk factors for violence against women in high-prevalence settings: A mixed methods systematic review and meta-synthesis

**Database**	**Search string** (second round search highlighted)	**Results (#)**
	abuse" OR married OR religion OR "male authority" OR disability OR "mental health" OR conflict OR poverty OR "gender inequality" OR "microcredit participation" OR "HIV status" OR "witnessing violence" OR "natural disaster" OR "food insecurity" OR parity OR "sex work" OR infidelity OR polygamy OR "duration of marriage" OR income OR dowry OR autonomy OR empowerment OR "circumstances of first sex" OR "media exposure" OR "peer network")<br>(2) (“violence against women” or “gender-based violence” or “gender violence”) AND ("intimate partner violence" OR "domestic violence" OR "family violence" OR "partner abuse" OR "spous* abuse" OR "battered women" OR maltreatment OR "marital rape" OR "forced sex" OR "non-partner sexual violence" OR "hono* killings" OR "forced marriage") AND ("risk marker" OR "risk factor" OR predictor OR correlate OR driver OR "socioeconomic factors" OR "social determinants" OR age OR alcohol OR "social norm" OR attitude OR employment OR "child abuse" OR married OR religion OR "male authority" OR disability OR "mental health" OR conflict OR poverty OR "gender inequality" OR "microcredit participation" OR "HIV status" OR "witnessing violence" OR "natural disaster" OR "food insecurity" OR parity OR "sex work" OR infidelity OR polygamy OR "duration of marriage" OR income OR dowry OR autonomy OR empowerment OR "circumstances of first sex" OR "media exposure" OR "peer network") AND (global or multinational or “multi country” or “cross national” or international)	
EMBASE / PSYINFO (Ovid)	(1) violence against women/ or gender-based violence/ or gender violence.mp. [mp=title, abstract, heading word, drug trade name, original title, device manufacturer, drug manufacturer, device trade name, keyword, floating subheading word, candidate term word]<br>(2) intimate partner violence/ or domestic violence/ or family violence/ or partner abuse/ or spous* abuse/ or battered women/ or maltreatment/ or marital rape/ or forced sex/ or non-partner sexual violence/ or hono* killings/ or forced marriage.mp. [mp=title, abstract, heading word, drug trade name, original title, device manufacturer, drug manufacturer, device trade name, keyword, floating subheading word, candidate term word]<br>(3) equatorial guinea/ or afghanistan/ or vanuatu/ or solomon islands/ or kiribati/ or democratic republic of the congo/ or liberia/ or timor-leste/ or gabon/ or cameroon/ or fiji/ or uganda/ or tanzania/ or burundi/ or sierra leone/ or bangladesh/ or bolivia/ or micronesia/ or zambia/ or sao tome/ or central african republic/ or tuvalu/ or angola.mp. [mp=title, abstract, heading word, drug trade name, original title, device manufacturer, drug manufacturer, device trade name, keyword, floating subheading word, candidate term word]<br>(4) risk marker/ or risk factor/ or predictor/ or correlate/ or driver/ or socioeconomic factors/ or social determinants/ or age/ or alcohol/ or social norm/ or attitude/ or employment/ or child abuse/ or married/ or religion/ or male authority/ or disability/ or mental health/ or conflict/ or poverty/ or gender inequality/ or microcredit participation/ or HIV status/ or witnessing violence/ or natural disaster/ or food insecurity/ or parity/ or sex work/ or infidelity/	EMBASE results = (2,3,4)346 + (1,2,4,5)8 = 354 [Search date 06/01/21]<br><br>PsychInfo results = (2,3,4)21 + (1,3,4,5)2 = 23 [Search date 06/01/21]

Mannell J, *et al. BMJ Global Health* 2022; 7:e007704. doi: 10.1136/bmjgh-2021-007704

Supplementary material:
Risk factors for violence against women in high-prevalence settings: A mixed methods systematic review and meta-synthesis

Database	Search string (second round search highlighted)	Results (#)
	or polygamy/ or duration of marriage/ or income/ or dowry/ or autonomy/ or empowerment/ or circumstances of first sex/ or media exposure/ or peer network.mp. [mp=title, abstract, heading word, drug trade name, original title, device manufacturer, drug manufacturer, device trade name, keyword, floating subheading word, candidate term word]<br>(5) global/ or multinational/ or multi country/ or cross national/ or international*.mp. [mp=title, abstract, heading word, drug trade name, original title, device manufacturer, drug manufacturer, device trade name, keyword, floating subheading word, candidate term word]	

Supplementary material:
Risk factors for violence against women in high-prevalence settings: A mixed methods systematic review and meta-synthesis

Table 3: Inclusion criteria

	**Inclusion criteria**	**Exclusion criteria**
**Population**: women survivors/ men as perpetrators	Individuals identifying as women (survivors of violence) or men (perpetrators of violence), including transgender women and men.	- Children or adolescents under 18 years old<br>- Male victims of violence
**Outcome**: violence against women	Violence against women as the outcome including "any act of gender-based violence that results in, or is likely to result in, physical, sexual or psychological harm or suffering to women, including threats of such acts, coercion or arbitrary deprivation of liberty, whether occurring in public or in private life."	- Mental health outcomes<br>- Human trafficking<br>- Modern slavery<br>- Female genital mutilation/cutting<br>- Child marriage
**Type of study**: reporting on primary data	Any publication reporting original data (peer reviewed, PhD theses or grey literature)	- Opinion pieces and editorials<br>- MSc dissertations/theses<br>- policy papers not reporting original data<br>- general reports
**Study location**: High prevalence countries	Global studies of violence against women without a focus on any specific country<br>Studies taking place in high prevalence countries including:<br>- Equatorial Guinea<br>- Afghanistan<br>- Vanuatu<br>- Solomon Islands<br>- Kiribati<br>- Democratic Republic of the Congo<br>- Liberia<br>- Timor-Leste<br>- Gabon<br>- Cameroon<br>- Fiji<br>- Uganda<br>- Tanzania<br>- Burundi<br>- Sierra Leone	Studies focused on countries not on the included country list

Supplementary material:
Risk factors for violence against women in high-prevalence settings: A mixed methods systematic review and meta-synthesis

	- Bangladesh<br>- Bolivia<br>- Micronesia<br>- Zambia<br>- Sao Tome and Principe<br>- Central African Republic<br>- Tuvalu<br>- Angola	

Mannell J, *et al. BMJ Global Health* 2022; 7:e007704. doi: 10.1136/bmjgh-2021-007704

Supplementary material:
Risk factors for violence against women in high-prevalence settings: A mixed methods systematic review and meta-synthesis

Table 4: Risk factor template

Risk factor	Concepts captured by term	Selection procedure
education	individual level of education; relative spousal education	Selected a-priori based on previous systematic reviews and search strategies.
age	individual age; relative spousal age; age at marriage	
alcohol	individual alcohol (mis)use; alcohol use before sex; alcohol use before/during violence	
social norm	social norms related to the use of violence and gender roles	
attitude	justification, normalisation and acceptance of violence	
substance use	individual substance (mis)use; substance use before sex; substance use before violence	
employment status	individual employment status; unemployment; job type	
child abuse	experiences of any type of abuse during childhood	
ethnicity	identification with an ethnic group; discrimination based on ethnic group identification	
married	marital status (married, single, cohabiting, informal relationship, divorced, widowed)	
religion	identification with a religious group; discrimination based on religious group identification	
male authority	male (partner, in-laws, other family members, manager, client) controlling behaviour	
disability	disability status; disability stigma and discrimination	
mental health	mental health illnesses (e.g. depression, anxiety, PTSD); mental health stigma and discrimination	
armed conflict	residing in conflict zone; experience of violence during conflict; participation in violence during conflict; displacement due to conflict	
poverty	low socioeconomic status; wealth index	
gender inequality	unequal gender norms; gender disparities (e.g. income, education); sexism and discrimination	
microcredit participation	participation in microcredit programmes; NGO membership	Iteratively added to the risk factor template based on screening of papers retrieved from the first round of searching. Risk factors were labelled based on database searching to determine most commonly used term for each risk factor, also considering
HIV status	HIV status discordance; HIV seropositivity; HIV stigma and discrimination	
witnessing violence	witnessing parental IPV; witnessing violence during conflict; witnessing environmental violence	
natural disaster	environmental shocks (e.g. draught, cyclone)	
food insecurity	experiences of lack of food and hunger	
parity	number of children	
sex work	transactional sex; sex work stigma and discrimination	
infidelity	extra-marital sex; adultery	
polygamy	polygyny; multiple wives/partners; experience of being a co-wife	
duration of marriage	length of marriage/relationship	
income	individual income level; relative spousal income	
dowry	dowry practices; bride price practices	
autonomy	women's decision-making power	
empowerment	women's empowerment (including economic empowerment)	

Supplementary material:
Risk factors for violence against women in high-prevalence settings: A mixed methods systematic review and meta-synthesis

circumstances of first sex	forced first sex; age at sexual initiation	MeSH terms.
media exposure	TV/radio/phone ownership; reads newspaper	
peer network	peer group influence; peer network norms	

Supplementary material:
Risk factors for violence against women in high-prevalence settings: A mixed methods systematic review and meta-synthesis

Table 5: Criteria for assessing fatal flaws in study design

**Criteria for assessing fatal flaws across study designs**	
Are the aims and objectives of the research clearly stated?	☐
Is the research design clearly specified and appropriate for the aims and objectives of the research?	☐
Do the researchers provide a clear account of the process by which their findings were reproduced?	☐
Do the researchers display enough data to support their interpretations and conclusions?	☐
Is the method of analysis appropriate and adequately explicated?	☐
Additional comments:	

Reference: Dixon-Woods, M., Cavers, D., Agarwal, S., Annandale, E., Arthur, A., Harvey, J., Hsu, R., Katbamna, S., Olsen, R., Smith, L. and Riley, R., 2006. Conducting a critical interpretive synthesis of the literature on access to healthcare by vulnerable groups. *BMC medical research methodology*, *6*(1), pp.1-13.

Supplementary material:
Risk factors for violence against women in high-prevalence settings: A mixed methods systematic review and meta-synthesis

Table 6: All included records, by study

Study name/ design	Reference	Focus	VAW type(s)	Perpetrator(s)	Highest level of analysis	Sample
**Afghanistan**						
**DHS 2015** Quantitative; cross-sectional	Qamar, M., Harris, M. A., & Tustin, J. L. (2020). The Association Between Child Marriage and Domestic Violence in Afghanistan. *Journal of Interpersonal Violence,* online.	Child marriage and IPV experience among women.	Physical, sexual, psychological	Partner	Individual/ relational	Women n= 21,324
**Women for Women Trial** Quantitative; cross-sectional	Jewkes, R., Corboz, J., & Gibbs, A. (2019). Violence against Afghan women by husbands, mothers-in-law and siblings-in-law/siblings: Risk markers and health consequences in an analysis of the baseline of a randomised controlled trial. *PLoS ONE*, *14*(2).	Risk factors for domestic violence among women.	Physical	Partner, family member	Individual/ relational	Women n= 1,436
	Gibbs, A., Corboz, J., & Jewkes, R. (2018). Factors associated with recent intimate partner violence experience amongst currently married women in Afghanistan and health impacts of IPV: A cross sectional study. *BMC Public Health*, *18*(1).	Risk factors for IPV among married women.	Physical, psychological	Partner	Individual/ relational	
	Jewkes, R., Corboz, J., & Gibbs, A. (2018). Trauma exposure and IPV experienced by Afghan women: Analysis of the baseline of a randomised controlled trial. *PLoS ONE*, *13*(10).	Trauma exposure and IPV experience among women.	Physical	Partner, family member	Individual/ relational	
Qualitative; in-depth interviews	Mannell, J., Grewal, G., Ahmad, L., & Ahmad, A. (2020). A Qualitative Study of Women's Lived Experiences of Conflict and Domestic Violence in Afghanistan. *Violence Against Women*. Online.	Women's lived experiences of conflict and domestic violence.	Physical, psychological	Partner		Women n= 20
Qualitative; in-depth interviews	Ahmad, L., & Anctil Avoine, P. (2018). Misogyny in 'post-war' Afghanistan: the changing frames of sexual and gender-based violence. *Journal of Gender Studies*, *27*(1), 86–101.	Misogyny and gender-based violence in post-war Afghanistan.	Physical, sexual, psychological	Partner, family member, stranger		Women n= 40
**Angola**						
**Cooperation on Research and Development in Angola** Qualitative; focus group discussions	Strønen, I., Nangacovie, M., (2016). *Violence against women in the urban poverty of Angola*. Chr. Michelsen Institute. Brief volume 15, no. 16).	Violence against women in the context of urban poverty in Angola.	Physical, sexual	Partner		Women n= 20
**DHS 2016** Quantitative; cross-sectional	Yaya, S., Kunnuji, M., & Bishwajit, G. (2019). Intimate Partner Violence: A Potential Challenge for Women's Health in Angola. *Challenges*, *10*(1), 21.	Risk factors for IPV experience among women.	Physical, sexual, psychological	Partner	Individual/ relational	Women n= 7,699
	Yaya, S., & Ghose, B. (2019). Alcohol Drinking by Husbands/Partners Is Associated with Higher Intimate Partner	Partner's alcohol use and IPV among	Physical, sexual,	Partner	Individual/ relational	

Supplementary material:
Risk factors for violence against women in high-prevalence settings: A mixed methods systematic review and meta-synthesis

Study name/ design	Reference	Focus	VAW type(s)	Perpetrator(s)	Highest level of analysis	Sample
	Violence against Women in Angola. *Safety*, *5*(5).	women.	psychological			
Quantitative; cross-sectional	Nimi, T., Fraga, S., Costa, D., et al. (2019). Prevalence, determinants, and effects of violence during pregnancy: A maternity-based cross-sectional study in Luanda, Angola. *Journal of Public Health in Africa*, *10*(2), 116–122.	Prevalence and risk factors for violence among pregnant women.	Physical, psychological	Partner, family member	Individual/ relational	Women n= 995
**Bangladesh**						
**DHS 2003** Quantitative; cross-sectional	Johnson, K. B., & Das, M. B. (2009). Spousal violence in Bangladesh as reported by men: Prevalence and risk factors. *Journal of Interpersonal Violence*, *24*(6), 977–995.	Prevalence and risk factors for IPV perpetration among men.	Physical	Partner	Individual/ relational	Men n= 4,297
	Aklimunnessa, K., Khan, M. M. H., Kabir, M., & Mori, M. (2007). Prevalence and correlates of domestic violence by husbands against wives in Bangladesh: evidence from a national survey. *The Journal of Men's Health & Gender*, *4*(1), 52–63.	Prevalence and risk factors for IPV perpetration among men.	Physical, sexual	Partner	Individual/ relational	
	Silverman, J. G., Decker, M. R., Kapur, N. A., et al. (2006). Violence against wives, sexual risk and sexually transmitted infection among Bangladeshi men. *Sexually Transmitted Infections*, *83*(3), 211–215.	Men's sexual behaviours and IPV perpetration.	Physical, sexual	Partner	Individual/ relational	
**DHS 2007** Quantitative; cross-sectional	Afiaz, A., Biswas, R. K., Shamma, R., & Ananna, N. (2020). Intimate partner violence (IPV) with miscarriages, stillbirths and abortions: Identifying vulnerable households for women in Bangladesh. *PLoS ONE*, *15*(7).	Risk factors for IPV among women and association with reproductive health outcomes.	Physical	Partner	Individual/ relational	Women n= 4,489 Men n= 3,339
	Sanawar, S. B., Islam, M. A., Majumder, S., & Misu, F. (2019). Women's empowerment and intimate partner violence in Bangladesh: investigating the complex relationship. *Journal of Biosocial Science,* 51(2), 188–202.	Relationship between women's empowerment and IPV.	Physical, sexual	Partner	Individual/ relational	
	Murshid, N. S., & Murshid, N. (2018). Intergenerational Transmission of Marital Violence: Results From a Nationally Representative Sample of Men. *Journal of Interpersonal Violence*, *33*(2), 211–227.	Intergenerational transmission of IPV among men.	Partner	Partner	Individual/ relational	
	Yount, K. M., Roof, K. A., & Naved, R. T. (2018). Multilevel influences on men's partner violence justification, control over family decisions, and partner violence perpetration in Bangladesh. *Psychology of Violence*, *8*(3), 367–378.	Influences on men's IPV perpetration.	Physical	Partner	Community	
	Islam, M. J., Rahman, M., Broidy, L., Haque, et al. (2017). Assessing the link between witnessing inter-parental violence and the perpetration of intimate partner violence in	Intergenerational transmission of IPV among men.	Physical, sexual	Partner	Individual/ relational	

Supplementary material:
Risk factors for violence against women in high-prevalence settings: A mixed methods systematic review and meta-synthesis

Study name/ design	Reference	Focus	VAW type(s)	Perpetrator(s)	Highest level of analysis	Sample
	Bangladesh. *BMC Public Health*, *17*(1).					
	Murshid, N. S. (2017). Men's Report of Domestic Violence Perpetration in Bangladesh: Correlates From a Nationally Representative Survey. *Journal of Interpersonal Violence*, *32*(2), 290–307.	Risk factors for men's IPV perpetration.	Physical, sexual	Partner	Individual/ relational	
	Akhter, R., & Wilson, J. K. (2016). Using an Ecological Framework to Understand Men's Reasons for Spousal Abuse: An Investigation of the Bangladesh Demographic and Health Survey 2007. *Journal of Family Violence*, *31*(1), 27–38.	Risk factors for IPV perpetration among men.	Physical	Partner	Individual/ relational	
	Murshid, N. S. (2016). Men's response to their wives' participation in microfinance: perpetration and justification of intimate partner violence in Bangladesh. *Public Health, 141, 146-152*.	Wife's microfinance participation and male perpetration of IPV.	Physical, sexual	Partner	Individual/ relational	
	Murshid, N. S., Akincigil, A., & Zippay, A. (2015). Microfinance Participation and Domestic Violence in Bangladesh: Results From a Nationally Representative Survey. *Journal of Interpersonal Violence*, *31*(9), 1579–1596.	Wife's microfinance participant and female experience of IPV.	Physical, sexual	Partner	Individual/ relational	
	Islam, T. M., Tareque, M. I., Sugawa, M., & Kawahara, K. (2015). Correlates of Intimate Partner Violence Against Women in Bangladesh. *Journal of Family Violence*, *30*(4), 433–444.	Risk factors for women's experience of IPV.	Physical, sexual	Partner	Individual/ relational	
	Islam, T. M., Tareque, I., Tiedt, A. D., & Hoque, N. (2014). The intergenerational transmission of intimate partner violence in Bangladesh. *Global Health Action*, *7*(1).	Intergenerational transmission of IPV among women.	Physical, sexual	Partner	Individual/ relational	
	Rahman, M., Hoque, M. A., Mostofa, M. G., & Makinoda, S. (2014). Association between adolescent marriage and intimate partner violence: A study of young adult women in Bangladesh. *Asia-Pacific Journal of Public Health*, *26*(2), 160–168.	Adolescent marriage and IPV among women.	Physical, sexual	Partner	Individual/ relational	
	Bajracharya, A., & Amin, S. (2013). Microcredit and Domestic Violence in Bangladesh: An Exploration of Selection Bias Influences. *Demography*, *50*(5), 1819–1843.	Microcredit participation and IPV.	Physical	Partner	Individual/ relational	
	Dalal, K., Dahlström, Ö., & Timpka, T. (2013). Interactions between microfinance programmes and non-economic empowerment of women associated with intimate partner violence in Bangladesh: a cross-sectional study. *BMJ Open*, *3*(12).	Microfinance, non-economic empowerment and IPV among women.	Physical, sexual	Partner	Individual/ relational	
	Department for International Development. (2013). From Evidence to Policy: Addressing Gender-Based Violence	Risk factors for IPV.	Physical, sexual	Partner	Community	

Supplementary material:
Risk factors for violence against women in high-prevalence settings: A mixed methods systematic review and meta-synthesis

Study name/ design	Reference	Focus	VAW type(s)	Perpetrator(s)	Highest level of analysis	Sample
	against Women and Girls in Bangladesh. London, UK.					
	Rahman, M., Nakamura, K., Seino, K., & Kizuki, M. (2013). Does gender inequity increase the risk of intimate partner violence among women? Evidence from a national Bangladeshi sample. *PLoS ONE*, *8*(12).	Gender inequity and IPV experience among women.	Physical, sexual	Partner	Individual/relational	
	Rapp, D., Zoch, B., Khan, M. M. H., Pollmann, T., & Krämer, A. (2012). Association between gap in spousal education and domestic violence in India and Bangladesh. *BMC Public Health*, *12*(1).	Spousal education gap and IPV.	Physical	Partner	Individual/relational	
	Rahman, M., Hoque, M. A., & Makinoda, S. (2011). Intimate Partner Violence Against Women: Is Women Empowerment a Reducing Factor? A Study from a National Bangladeshi Sample. *Journal of Family Violence*, *26*(5), 411–420.	Women's empowerment and IPV experience.	Physical, sexual	Partner	Individual/relational	
	De, P. K., & Christian, A. (2020). Microfinance participation and intimate partner violence among women in Bangladesh. *Oxford Economic Papers*, *72*(2), 435–452.	Microfinance participation and IPV among women.	Physical	Partner	Individual/relational	Women n= 12,662<br>Men n= 9,847 (urban health survey dataset)
	Sambisa, W., Angeles, G., Lance, P. M., Naved, R. T., & Thornton, J. (2011). Prevalence and correlates of physical spousal violence against women in slum and nonslum areas of urban Bangladesh. *Journal of Interpersonal Violence*, *26*(13), 2592–2618.	Prevalence and risk factors for IPV experience among urban women.	Physical	Partner	Individual/relational	
	Sambisa, W., Angeles, G., Lance, P. M., Naved, R. T., & Curtis, S. L. (2010). Physical and Sexual Abuse of Wives in Urban Bangladesh: Husbands' Reports. *Studies in Family Planning*, *41*(3), 165–178.	Risk factors for IPV perpetration among men.	Physical, sexual	Partner	Individual/ relational	
**BRISC trial 2020** Quantitative; cross-sectional and longitudinal	Hamadani, J. D., Hasan, M. I., Baldi, A. J., et al. (2020). Immediate impact of stay-at-home orders to control COVID-19 transmission on socioeconomic conditions, food insecurity, mental health, and intimate partner violence in Bangladeshi women and their families: an interrupted time series. *The Lancet Global Health*, *8*(11), e1380–e1389.	Impact of covid-19 lockdown orders on IPV (among other outcomes).	Physical, sexual, psychological	Partner	Individual/ relational	Women n= 2,424
**Gender Ideology, Microcredit & Marital Violence in Rural Bangladesh 2009** Quantitative; cross-sectional	Karim, K. M. R., & Law, C. K. (2016). Microcredit and Marital Violence: Moderating Effects of Husbands' Gender Ideology. *Journal of Family Violence*, *31*(2), 227–238.	Microcredit, gender ideology and IPV perpetration among men.	Physical, sexual, psychological	Partner	Individual/ relational	Men n= 243
**HERrespect**	Gibbs, A., Jewkes, R., Willan, S., et al. (2019). Workplace	Risk factors for	Physical,	Manager	Individual/	Women n=

Supplementary material:
Risk factors for violence against women in high-prevalence settings: A mixed methods systematic review and meta-synthesis

Study name/ design	Reference	Focus	VAW type(s)	Perpetrator(s)	Highest level of analysis	Sample
**Intervention 2016** Quantitative; cross-sectional	violence in Bangladesh's garment industry. *Social Science and Medicine*, *235*.	workplace violence among female garment workers.	psychological		relational	800 Men n= 395
	Naved, R. T., al Mamun, M., Parvin, K., et al. (2018). Magnitude and correlates of intimate partner violence against female garment workers from selected factories in Bangladesh. *PLoS ONE*, *13*(11).	Prevalence and risk factors for IPV experience among female garment workers.	Physical, sexual, economic	Partner	Individual/ relational	
**Healthy Fertility Study (HFS) 2007** Quantitative; cross-sectional	Stake, S., Ahmed, S., Tol, W., et al. (2020). Prevalence, associated factors, and disclosure of intimate partner violence among mothers in rural Bangladesh. *Journal of Health, Population and Nutrition*, *39*(1).	Prevalence and risk factors for IPV experience among mothers.	Physical, sexual	Partner	Individual/ relational	Women n= 3,966
***Suchana* 2016 – 2017** Quantitative; cross-sectional	Haque, M. A., Choudhury, N., Ahmed, S. M. T et al. (2020). Factors Associated with Domestic Violence in Rural Bangladesh. *Journal of Interpersonal Violence*. Online.	Risk factors for domestic violence.	Physical, psychological	Partner, family member	Individual/ relational	Women n= 5,440
**UN Multi-Country Study on Men and Violence 2011** Quantitative; cross-sectional	Semenza, D. C., Roof, K. A., James-Hawkins, L., Cheong, Y. F., Naved, R. T., & Yount, K. M. (2019). Gender-Equitable Parental Decision Making and Intimate Partner Violence Perpetration in Bangladesh. *Journal of Marriage and Family*, *81*(4), 920–935.	Gender-equitable parental decision-making and IPV perpetration among men.	Physical	Partner	Community	Men n= 2,400
	James-Hawkins, L., Cheong, Y. F., Naved, R. T., & Yount, K. M. (2018). Gender norms, violence in childhood, and men's coercive control in marriage: A multilevel analysis of young men in Bangladesh. *Psychology of Violence*, *8*(5), 580–595.	Gender norms, childhood violence and controlling behaviour in marriage among men.	Controlling behaviour	Partner	Community	
	Yount, K. M., James-Hawkins, L., Cheong, Y. F., & Naved, R. T. (2018). Men's perpetration of partner violence in Bangladesh: Community gender norms and violence in childhood. *Psychology of Men and Masculinity*, *19*(1), 117–130.	Community gender norms, childhood violence and IPV perpetration among men.	Physical	Partner	Community	
Quantitative; cross-sectional	Aktaruzzaman, K., & Farooq, O. (2020). Microfinance and domestic violence. *Journal of Adult Protection*, *22*(5).	Microfinance and IPV experience among women.	Physical, psychological	Partner	Individual/ relational	Women n= 1,037
	Reiss, K., Andersen, K., Pearson, E., et al. (2019). Unintended consequences of mHealth interactive voice messages promoting contraceptive use after menstrual regulation in Bangladesh: Intimate partner violence results from a	mHealth intervention promoting contraceptive use and IPV.	Physical	Partner	Individual/ relational	Women n= 772

Supplementary material:
Risk factors for violence against women in high-prevalence settings: A mixed methods systematic review and meta-synthesis

Study name/ design	Reference	Focus	VAW type(s)	Perpetrator(s)	Highest level of analysis	Sample
	randomized controlled trial. *Global Health Science and Practice*, *7*(3), 386–403.					
	Chowdhury, M. A. K., Rahman, A. E., Morium, S., et al. (2018). Domestic Violence Against Women in Urban Slums of Bangladesh: A Cross-Sectional Survey. *Journal of Interpersonal Violence*, online.	Risk factors for IPV experience among women.	Physical	Partner	Individual/ relational	Women n= 87
	Karim, R., & Swahnberg, K. (2018). Does Female Authority Prevent Male Marital Violence? Evidence From Rural Bangladesh. *Journal of Interpersonal Violence*. Online.	Female authority and IPV perpetration among men.	Physical, sexual, psychological	Partner	Individual/ relational	Men n= 342
	Schuler, S. R., & Nazneen, S. (2018). Does Intimate Partner Violence Decline as Women's Empowerment becomes Normative? Perspectives of Bangladeshi Women. *World Development*, *101*, 284–292.	Women's empowerment and IPV experience.	Physical	Partner	Individual/ relational	Women n= 74
	Islam, M. J., Mazerolle, P., Broidy, L., & Baird, K. (2017). Exploring the Prevalence and Correlates Associated With Intimate Partner Violence During Pregnancy in Bangladesh. *Journal of Interpersonal Violence*, online.	Prevalence and risk factors for IPV among pregnant women.	Physical, sexual, psychological	Partner	Individual/ relational	Women n= 426
	Yount, K. M., Crandall, A. A., Cheong, Y. F., et al. (2016). Child Marriage and Intimate Partner Violence in Rural Bangladesh: A Longitudinal Multilevel Analysis. *Demography*, *53*(6), 1821–1852.	Child marriage and IPV experience among women.	Physical	Partner	Community	Women n= 3,355
	Hasan, T., Muhaddes, T., Camellia, S., Selim, N., & Rashid, S. F. (2014). Prevalence and Experiences of Intimate Partner Violence Against Women With Disabilities in Bangladesh: Results of an Explanatory Sequential Mixed-Method Study. *Journal of Interpersonal Violence*, *29*(17), 3105–3126.	IPV among women with disabilities.	Physical, sexual, psychological	Partner	Individual/ relational	Women n= 226
	Heath, R. (2014). Women's Access to Labor Market Opportunities, Control of Household Resources, and Domestic Violence: Evidence from Bangladesh. *World Development*, *57*, 32–46.	Women's access to labour market opportunities, control of household resources and IPV.	Physical	Partner	Individual/ relational	Women n= 1,395
	Dalal, K., Rahman, F., & Jansson, B. (2009). Wife abuse in rural Bangladesh. *Journal of Biosocial Science*, *41*(5), 561–573.	Risk factors for IPV among women in Bangladesh.	Physical, psychological, controlling behaviours	Partner	Individual/ relational	Women n= 4,411
	Bhuiya, A., Sharmin, T., & Hanifi, M. A. (2003). Nature of domestic violence against women in a rural area of Bangladesh: Implication for preventive Interventions. *Journal of Health Population and Nutrition*, *21*(1), 48–54.	Risk factors for VAW among couples.	Physical, psychological	Partner	Individual/ relational	Women n= 19

Supplementary material:
Risk factors for violence against women in high-prevalence settings: A mixed methods systematic review and meta-synthesis

Study name/ design	Reference	Focus	VAW type(s)	Perpetrator(s)	Highest level of analysis	Sample
Qualitative; in-depth interviews	Schuler, S. R., Lenzi, R., Badal, S. H., & Bates, L. M. (2017). Women's Empowerment as a Protective Factor Against Intimate Partner Violence in Bangladesh: A Qualitative Exploration of the Process and Limitations of Its Influence. *Violence Against Women*, *23*(9), 1100–1121.	Women's perspectives on IPV and empowerment.	Physical	Partner		Women n= 74
	Murshid, N. S., & Zippay, A. (2017). Microfinance Participation and Marital Violence in Bangladesh: A Qualitative Inquiry. *Violence Against Women*, *23*(14), 1752–1770.	Microfinance and IPV in marriage.	Physical, psychological	Partner		Women n= 30
	Das, T. K., Alam, Md. F., Bhattacharyya, R., & Pervin, A. (2015). Causes and Contexts of Domestic Violence: Tales of Help-Seeking Married Women in Sylhet, Bangladesh. *Asian Social Work and Policy Review*, *9*(2), 163–176.	Stories of help-seeking among married women experiencing IPV.	Physical, psychological, controlling behaviours, economic	Partner		Women n= 42
	Islam, Md. M., & Karim, K. M. R. (2012). Men's Views on Gender and Sexuality in a Bangladesh Village. *International Quarterly of Community Health Education*, *32*(4), 339–354.	Men's views on gender and sexuality.	Physical, sexual	Partner		Men n= 10
	Naved, R. T., Blum, L. S., Chowdhury, et al. (2012). Violence against women with chronic maternal disabilities in rural Bangladesh. *Journal of Health, Population and Nutrition*, *30*(2), 181–192.	Violence against women with chronic maternal disabilities.	Physical, sexual, psychological	Partner, family member		Women n= 17
	Karim, K. M. R. (2006). Men's Arrack Drinking and Domestic Violence against Women in a Bangladeshi Village. *International Quarterly of Community Health Education*, *25*(4), 367–380.	Men's arrack drinking and domestic violence.	Physical, sexual, psychological	Partner		Women n= 50<br>Men n= 50
	Khan, M. E., Townsend, J. W., & D'Costa, S. (2002). Behind closed doors: A qualitative study of sexual behaviour of married women in Bangladesh. *Culture, Health & Sexuality*, *4*(2), 237–256.	Qualitative study of sexual behaviour of married women.	Physical, sexual	Partner		Women n= 54
Qualitative; combination of methods	Akhter, R., Wilson, J. K., Haque, S. E., & Ahamed, N. (2020). Like a Caged Bird: The Coping Strategies of Economically Empowered Women Who Are Victims of Intimate Partner Violence in Bangladesh. *Journal of Interpersonal Violence*. Online.	Coping strategies among economically empowered women who experience IPV.	Physical	Partner		Women n= 19<br>Key informants n= 8
	Samuels, F., le Masson, V., & Gupta, T. (2019). One Step Forwards half a Step Backwards: Changing Patterns of Intimate Partner Violence in Bangladesh. *Journal of Family Violence*, *34*(2), 107–118.	Changing patterns of intimate partner violence in Bangladesh.	Physical, psychological, sexual, controlling behaviours, economic	Partner		Women n= 6<br>Men n= 17<br>Key informants n= 40

Supplementary material:
Risk factors for violence against women in high-prevalence settings: A mixed methods systematic review and meta-synthesis

Study name/ design	Reference	Focus	VAW type(s)	Perpetrator(s)	Highest level of analysis	Sample
	Islam, M. M., Jahan, N., & Hossain, M. D. (2018). Violence against women and mental disorder: a qualitative study in Bangladesh. *Tropical Medicine and Health*, *46*(1), 5.	Violence against women and mental disorder.	Physical, sexual, psychological, controlling behaviours, economic	Partner, family members		Women n= 19 Key informants n= 7
	Naved, R., Rahman, T., Willan, S., et al. (2018). Female garment workers' experiences of violence in their homes and workplaces in Bangladesh: A qualitative study. *Social Science & Medicine*, *196*, 150–157.	Female garment workers' experiences of IPV and workplace violence.	Physical, sexual, psychological, economic	Partner, manager		Women n= 41
	Schuler, S. R., Lenzi, R., Huda Badal, S., & Nazneen, S. (2017). Men's perspectives on women's empowerment and intimate partner violence in rural Bangladesh. *Culture, Health & Sexuality*, *20*(1), 113–127.	Men's perspectives on women's empowerment and IPV.	Physical	Partner		Women n= 74
	Schuler, S. R., Lenzi, R., Badal, S. H., & Bates, L. M. (2017). Women's Empowerment as a Protective Factor Against Intimate Partner Violence in Bangladesh: A Qualitative Exploration of the Process and Limitations of Its Influence. *Violence Against Women*, *23*(9), 1100–1121.	Women's empowerment as protection against IPV.	Physical	Partner		Women n= 61 Men n= 46
	Anwary, A. (2015). Construction of hegemonic masculinity: Violence against wives in Bangladesh. *Women's Studies International Forum*, *50*, 37–46.	Hegemonic masculinity and IPV.	Physical	Partner		Not specified
	Schuler, S. R., Lenzi, R., Nazneen, S., & Bates, L. M. (2013). Perceived Decline in Intimate Partner Violence Against Women in Bangladesh: Qualitative Evidence. *Studies in Family Planning*, *44*(3), 243–257.	Perceived decline in IPV against women in Bangladesh.	Physical	Partner		Women n= 62 Men n= 16 FGDs n= 11 (breakdown not available)
Qualitative; case study	Rezwana, N., & Pain, R. (2020). Gender- based violence before, during and after cyclones: slow violence and layered disasters. *Disasters*. 45, 741-761.	Gender-based violence before, during and after cyclones.	Physical, sexual, psychological	Partner, family member, stranger		Women n= 29 Men n= 8 Key informants n=8
Mixed methods	Karim, K. M. R., Emmelin, M., Resurreccion, B. P., & Wamala, S. (2012). *International Water Development Projects and Marital Violence: Experiences From Rural Bangladesh*.	Water development projects and IPV.	Physical	Partner		Women n= 16 Key informants n=5
	Schuler, S. R., & Islam, F. (2008). Women's Acceptance of Intimate Partner Violence within Marriage in Rural Bangladesh. *Studies in Family Planning*, *39*(1), 49–58.	Women's acceptance of IPV in marriage.	Physical	Partner		Women n= 606
	Naved, R. T., & Persson, L. Å. (2005). Factors Associated with Spousal Physical Violence against Women in Bangladesh.	Risk factors for IPV among women.	Physical	Partner		Women n= 2,730

Mannell J, *et al. BMJ Global Health* 2022; 7:e007704. doi: 10.1136/bmjgh-2021-007704

Supplementary material:
Risk factors for violence against women in high-prevalence settings: A mixed methods systematic review and meta-synthesis

Study name/ design	Reference	Focus	VAW type(s)	Perpetrator(s)	Highest level of analysis	Sample
	Family Planning, 36(4).					
	Bates, L. M., Schuler, S. R., Islam, F., & Islam, M. K. (2004). Socioeconomic Factors and Processes Associated With Domestic Violence in Rural Bangladesh. *International Family Planning Perspectives*, *30*(04), 190–199.	Socioeconomic factors associated with IPV.	Physical	Partner		Women n= 1,276
**WHO Multi-Country Study on Women's Health and Domestic Violence against Women 2000 – 2004** Quantitative; cross-sectional	VanderEnde, K. E., Sibley, L. M., Cheong, Y. F., et al. (2015). Community Economic Status and Intimate Partner Violence Against Women in Bangladesh: Compositional or Contextual Effects? *Violence Against Women*, *21*(6), 679–699.	Community economic status and IPV among women.	Physical, sexual	Partner	Individual/ relational	Women n= 3,130
	Naved, R. T. (2013). Sexual violence towards married women in Bangladesh. *Archives of Sexual Behavior*, *42*(4), 595–602.	Risk factors for sexual IPV experience among women.	Sexual	Partner	Individual/ relational	
	Naved, R. T., & Persson, L. Å. (2008). Factors associated with physical spousal abuse of women during pregnancy in Bangladesh. *International Family Planning Perspectives*, *34*(2), 71–78.	Risk factors for physical IPV during pregnancy.	Physical	Partner	Individual/ relational	
**Bolivia**						
**Bolivia DHS 2008** Quantitative; cross-sectional	Camargo, E. (2019). Gender inequality and intimate partner violence in Bolivia. *Revista Colombiana de Sociologia*, *42*(2), 257–277.	Gender inequality and women's experience of IPV.	Physical, psychological	Partner	Individual/ relational	Women n= 10,188 Men n= 2,795
	Meekers, D., Pallin, S. C., & Hutchinson, P. (2013). Prevalence and correlates of physical, psychological, and sexual intimate partner violence in Bolivia. *Global Public Health*, *8*(5), 588–606.	Prevalence and risk factors for IPV among women.	Physical, sexual, psychological	Partner	Individual/ relational	
Quantitative; cross-sectional	Stieglitz, J., Trumble, B. C., Kaplan, H., & Gurven, M. (2018). Marital violence and fertility in a relatively egalitarian high-fertility population. *Nature Human Behaviour*, *2*(8), 565–572.	Exploring marital violence and fertility among Tsimané forager-horticulturists.	Physical	Partner	Individual/ relational	Women n= 105
	Stieglitz, J., Gurven, M., Kaplan, H., & Winking, J. (2012). Infidelity, jealousy, and wife abuse among Tsimane forager–farmers: testing evolutionary hypotheses of marital conflict. *Evolution and Human Behavior*, *33*(5), 438–448.	Infidelity, jealous and IPV among Tsimané forager horticulturists.	Not specified	Partner	Individual/ relational	Women n= 25 Men n= 21
	Stieglitz, J., Kaplan, H., Gurven, M., et al. (2011). Spousal violence and paternal disinvestment among Tsimane' forager-horticulturalists. *American Journal of Human Biology*, *23*(4), 445–457.	IPV and paternal disinvestment among Tsimané forager horticulturists.	Physical	Partner	Individual/ relational	Women n= 49
**Burundi**						
Quantitative;	Nandi, C., Elbert, T., Bambonye, M., Weierstall, R., Reichert,	IPV perpetration	Physical,	Partner	Individual/	Men n= 381

Supplementary material:
Risk factors for violence against women in high-prevalence settings: A mixed methods systematic review and meta-synthesis

Study name/ design	Reference	Focus	VAW type(s)	Perpetrator(s)	Highest level of analysis	Sample
cross-sectional	M., Zeller, A., & Crombach, A. (2017). Predicting domestic and community violence by soldiers living in a conflict region. *Psychological Trauma: Theory, Research, Practice, and Policy*, *9*(6), 663–671.	among soldiers in a conflict region.	sexual, psychological		relational	
	Crombach, A., & Bambonyé, M. (2015). Intergenerational violence in Burundi: Experienced childhood maltreatment increases the risk of abusive child rearing and intimate partner violence. *European Journal of Psychotraumatology*, *6*, 26995.	Intergenerational transmission of VAW among men and women.	Physical, sexual, controlling behaviours	Partner	Individual/ relational	Women n= 141<br>Men n= 141
**Cameroon**						
Quantitative; cross-sectional	Wadji, D. L., Ketcha Wanda, G. J. M., Wicky, C., et al. (2020). From the Mother to the Child: The Intergenerational Transmission of Experiences of Violence in Mother–Child Dyads Exposed to Intimate Partner Violence in Cameroon. *Journal of Interpersonal Violence*, online.	Intergenerational transmission of violence among women.	Physical, sexual	Partner	Individual/ relational	Women n=74
	Eze Eze, D. (2019). Microfinance programs and domestic violence in northern Cameroon; the case of the Familial Rural Income Improvement Program. *Review of Economics of the Household*, *17*(3), 947–967.	Microfinance programmes and IPV among women.	Physical, psychological	Partner	Individual/ relational	Women n= 320
	Parmar, P., Agrawal, P., Greenough, P. G., Goyal, R., & Kayden, S. (2012). Sexual violence among host and refugee population in Djohong District, Eastern Cameroon. *Global Public Health*, *7*(9), 974–994.	Risk factors for IPV among host and refugee populations.	Sexual	Partner, family member, stranger	Individual/ relational	Women n= 600
Qualitative; combination of methods	Lim, S., Peitzmeier, S., Cange, C., et al. (2015). Violence against female sex workers in Cameroon: Accounts of violence, harm reduction, and potential solutions. *Journal of Acquired Immune Deficiency Syndromes*, *68*, S241–S247.	Violence against female sex workers in Cameroon.	Physical, sexual	Client		Women n= 101
**Central African Republic**						
Qualitative; in-depth interviews	Human Rights Watch. (2017). *"They Said We Are Their Slaves." Sexual Violence by Armed Groups in the Central African Republic.* United States.	Sexual violence by armed groups.	Sexual	Combatants		Women n= 296
**Democratic Republic of the Congo**						
**DRC DHS 2007** Quantitative; cross-sectional	Tlapek, S. M. (2015). Women's Status and Intimate Partner Violence in the Democratic Republic of Congo. *Journal of Interpersonal Violence*, *30*(14), 2526–2540.	Women's status and experience of IPV.	Physical, sexual, psychological	Partner	Individual/ relational	Women n= 3,436
	Peterman, A., Palermo, T., & Bredenkamp, C. (2011). Estimates and determinants of sexual violence against women in the Democratic Republic of Congo. *American Journal of Public Health*, *101*(6), 1060–1067.	Risk factors for sexual violence among women.	Sexual	Partner, stranger	Individual/ relational	

Supplementary material:
Risk factors for violence against women in high-prevalence settings: A mixed methods systematic review and meta-synthesis

Study name/ design	Reference	Focus	VAW type(s)	Perpetrator(s)	Highest level of analysis	Sample
**DRC DHS 2014** Quantitative; cross-sectional	Tiruneh, F. N., Chuang, K.-Y., Ntenda, P. A. M., & Chuang, Y.-C. (2018). Unwanted pregnancy, pregnancy loss, and other risk factors for intimate partner violence in the Democratic Republic of the Congo. *Women & Health*, *58*(9), 983–1000.	Unwanted pregnancy, pregnancy loss and risk factors for IPV.	Physical, sexual, psychological	Partner	Community	Women n= 5,120
	Rustad, S. A., Østby, G., & Nordås, R. (2016). Artisanal mining, conflict, and sexual violence in Eastern DRC. *The Extractive Industries and Society*, *3*, 475–484. https://doi.org/10.1016/j.exis.2016.01.010	Artisanal mining, conflict and sexual violence among women.	Sexual	Partner, stranger	Individual/ relational	
**Engaging with Faith Groups to Prevent Violence Against Women and Girls in Conflict-affected Communities 2015 – 2017** Quantitative; cross-sectional	le Roux, E., Corboz, J., Scott, N., Sandilands, M., Lele, U. B., Bezzolato, E., & Jewkes, R. (2020). Engaging with faith groups to prevent VAWG in conflict-affected communities: Results from two community surveys in the DRC. *BMC International Health and Human Rights*, *20*(1).	Engaging with faith groups to prevent VAWG in conflict-affected communities.	Physical, sexual, psychological	Partner	Individual/ relational	Women n= 988 Men n= 961
**Pigs for Peace (livestock microfinance intervention)** Qualitative; in-depth interviews	Kohli, A., Perrin, N., Mpanano, R. M., et al. (2015). Family and community driven response to intimate partner violence in post-conflict settings. *Social Science & Medicine*, *146*, 276–284.	Family and community responses to IPV in post-conflict settings.	Physical, sexual	Partner		Women n= 13 Men n= 5
**Safe at Home 2018** Quantitative; cross-sectional	Scolese, A., Asghar, K., Pla Cordero, et al. (2020). Disability status and violence against women in the home in North Kivu, Democratic Republic of Congo. *Global Public Health*, *15*(7), 985–998.	Disability status and IPV experience among women.	Physical, sexual	Partner	Individual/ relational	Women n= 98
Quantitative; retrospective cohort	Bartels, S. A., Scott, J. A., Mukwege, D., Lipton, R. I., et al. (2010). Patterns of sexual violence in Eastern Democratic Republic of Congo: reports from survivors presenting to Panzi Hospital in 2006. *Conflict and Health*, *4*(1), 9.	Prevalence of sexual violence.	Sexual	Combatants	Individual/ relational	Women n= 1,021
Qualitative; in-depth interviews	Kiernan, B., Mishori, R., & Masoda, M. (2016). 'There is fear but there is no other work': a preliminary qualitative exploration of the experience of sex workers in eastern Democratic Republic of Congo. *Culture, Health & Sexuality*, *18*(3), 237–248.	Experiences of female sex-workers.	Physical, sexual	Clients		Women n= 8
	Elbert, T., Hinkel, H., Maedl, A., et al. (2013). *Sexual and Gender-Based Violence in the Kivu Provinces of the*	Insights from combatants on VAW.	Sexual	Stranger		Men n= 213

Supplementary material:
Risk factors for violence against women in high-prevalence settings: A mixed methods systematic review and meta-synthesis

Study name/ design	Reference	Focus	VAW type(s)	Perpetrator(s)	Highest level of analysis	Sample
	Democratic Republic of Congo: Insights from Former Combatants. World Bank, Washington, DC.					
	Human Rights Watch. (2009). Soldiers who rape, commanders who condone: Sexual violence and military reform int he Democratic Republic of Congo. New York, USA.	Sexual violence and military reform in the Democratic Republic of Congo.	Sexual	Partner, stranger		Women n= 36
Qualitative; focus group discussions	Kelly, J., Kabanga, J., Cragin, W., et al. (2012). 'If your husband doesn't humiliate you, other people won't': Gendered attitudes towards sexual violence in eastern Democratic Republic of Congo. *Global Public Health*, *7*(3), 285–298.	Gendered attitudes towards sexual violence in eastern DRC.	Sexual	Stranger		Women n= 45<br>Men n= 41
Qualitative; combination of methods	International Rescue Committee. (2019). *"Everything on her shoulders" Rapid assessment on gender and violence against women and girls in the Ebola outbreak in Beni, DRC*. New York, USA.	Gender and violence against women and girls in the Ebola outbreak	Physical, sexual, psychological	Partner, stranger		FGDs with men and women n= 34 (breakdown not available)<br>Key informants n= 24
**Fiji**						
Quantitative; cross-sectional	Fiji Women's Crisis Center. (2011). *Somebody's Life, Everybody's Business!* National Research on Women's Health and Life Experiences in Fiji (2010/2011): A survey exploring the prevalence, incidence and attitudes to intimate partner violence in Fiji. Suva, Fiji.	Risk factors for IPV experience among women.	Physical, sexual	Partner	Individual/ relational	Women n= 2,872
**Kiribati**						
**Kiribati Family Health and Support Study 2010**<br>Quantitative; cross-sectional	Secretariat of the Pacific Community. (2010). *Kiribati Family Health and Support Study: A study on violence against women and children*. Nouema, New Caledonia.	Risk factors for IPV experience among women.	Physical, sexual	Partner	Individual/ relational	
**Liberia**						
**DHS 2007**<br>Quantitative; cross-sectional	Kelly, J., Colantuoni, E., Robinson, C., & Decker, M. R. (2019). From political to personal violence: Links between conflict and non-partner physical violence in post-conflict Liberia. *Global Public Health*, *14*(12), 1639–1652.	Political conflict and non-partner physical violence experience among women.	Physical	Non-partner	Individual/ relational	Women n= 4,502
	Kelly, J. T. D., Colantuoni, E., Robinson, C., & Decker, M. R. (2018). From the battlefield to the bedroom: a multilevel analysis of the links between political conflict and intimate partner violence in Liberia. *BMJ Global Health*, *3*(2).	Political conflict and IPV experience among women.	Physical, sexual	Partner	Individual/ relational	

Supplementary material:
Risk factors for violence against women in high-prevalence settings: A mixed methods systematic review and meta-synthesis

Study name/ design	Reference	Focus	VAW type(s)	Perpetrator(s)	Highest level of analysis	Sample
Quantitative; cross-sectional	Sileo, K. M., Kershaw, T. S., Gilliam, et al (2019). Trauma Exposure and Intimate Partner Violence Among Young Pregnant Women in Liberia. *Journal of Interpersonal Violence*, online.	Trauma exposure and IPV experience among young pregnant women.	Physical, sexual, psychological	Partner	Individual/ relational	Women n= 183
	Allen, M., & Devitt, C. (2012). Intimate Partner Violence and Belief Systems in Liberia. *Journal of Interpersonal Violence*, *27*(17), 3514–3531.	Belief systems and IPV.	Physical, sexual, psychological, economic	Partner	Individual/ relational	Women n= 229
**Micronesia**						
**Family Health and Safety Study** Quantitative; cross-sectional)	FSM Department of Health and Social Affairs. (2014). *Federated States of Micronesia Family Health and Safety Study*. Palikir, FSM.	Risk factors for IPV experience among women.	Physical, sexual	Partner	Individual/r elational	Women n= 822
Qualitative; ethnography	Smith, S. A. (2019). Gender, Relationships and Sexual Violence in the Lives of Women from Chuuk, Micronesia. *Journal of Aggression, Maltreatment and Trauma*, *28*(2), 146–165.	Gender, relationships and sexual violence in the lives of women from Chuuk, Micronesia.	Sexual	Partner		Women n= 15
Mixed methods	Dugwen, G. L., Hancock, W. T., Gilmar, J., et al. (2013). Domestic violence against women on Yap, Federated States of Micronesia. *Hawai'i Journal of Medicine & Public Health, 72*(9), 318–322.	Prevalence and perceptions of IPV.	Physical, sexual	Partner		Women n= 8
**Sierra Leone**						
**Longitudinal Study of War-Affected youth in Sierra Leone (LSWAY) 2008** Quantitative; cross-sectional	Alleyne-Green, B., Kulick, A., Matsuzaka, S., & Betancourt, T. S. (2019). War Violence Exposure, Reintegration Experiences and Intimate Partner Violence Among a Sample of War-Affected Females in Sierra Leone. *Global Social Welfare*, *6*(2), 97–106.	War violence exposure, reintegration experiences and IPV among war-affected females.	Physical, sexual, psychological	Partner	Individual/ relational	Women n= 76 Men n= 92
	Alleyne-Green, B., Kulick, A., Grocher, K., & Betancourt, T. S. (2018). Physical and Sexual Violence Experienced by Male War-Affected Youth: Implications for Post-Conflict Functioning and Intimate Relationships. *Journal of Interpersonal Violence*. Online.	Experience of war-time violence and IPV among war-affected males.	Physical, sexual, psychological	Partner	Individual/ relational	
Quantitative; cross-sectional	Amowitz, L. L. (2002). Prevalence of War-Related Sexual Violence and Other Human Rights Abuses Among Internally Displaced Persons in Sierra Leone. *JAMA*, *287*(4), 513.	Prevalence of sexual violence among internally displaced persons.	Physical, sexual	Combatants	Individual/ relational	Women n= 994
Qualitative; in-	Human Rights Watch. (2009). "We'll Kill You If You Cry"	Sexual Violence in	Sexual	Partner,		Women n=

Supplementary material:
Risk factors for violence against women in high-prevalence settings: A mixed methods systematic review and meta-synthesis

Study name/ design	Reference	Focus	VAW type(s)	Perpetrator(s)	Highest level of analysis	Sample
depth interviews	Sexual Violence In The Sierra Leone Conflict. New York, USA.	the Sierra Leone Conflict.		stranger		300
**Solomon Islands**						
**Solomon Islands Family Health and Safety Study 2009** Quantitative; cross-sectional	Solomon Islands Ministry of Women Youth and Children's Affairs. (2009). *Solomon Islands Family Health and Safety Study: A study on violence against women and children*. Honiara, Solomon Islands.	Risk factors for IPV experience among women.	Physical, sexual	Partner	Individual/ relational	Women n= 2,246
**Tanzania**						
**Tanzania DHS 2010** Quantitative; cross-sectional	Vyas, S., & Heise, L. (2014). Using Propensity Score Matching to Estimate an "Unbiased Effect-Size" Between Women's Employment and Partner Violence in Tanzania. *Journal of Interpersonal Violence*, *29*(16), 2971–2990.	Propensity score matching to estimate effect-size between women's employment and IPV.	Physical, sexual, psychological	Partner	Individual/ relational	Women n= 5,688
	Vyas, S., & Heise, L. (2016). How do area-level socioeconomic status and gender norms affect partner violence against women? Evidence from Tanzania. *International Journal of Public Health*, *61*(8), 971–980.	Impact of area-level socioeconomic status and gender norms on IPV among women.	Physical, sexual	Partner	Community	
	Reese, B. M., Chen, M. S., Nekkanti, M., & Mulawa, M. I. (2017). Prevalence and Risk Factors of Women's Past-Year Physical IPV Perpetration and Victimization in Tanzania. *Journal of Interpersonal Violence*. Online.	Prevalence and risk factors for IPV among women.	Physical	Partner	Individual/ relational	
**Tanzania DHS 2015** Quantitative; cross-sectional	Vyas, S., & Jansen, H. A. F. M. (2018). Unequal power relations and partner violence against women in Tanzania: a cross-sectional analysis. *BMC Women's Health*, *18*(1), 185.	Unequal power relation and IPC among men and women.	Physical, sexual	Partner	Individual/ relational	Women n= 1,278 Men n= 1,278
**MAISHA trial** Quantitative; cross-sectional and longitudinal	Abramsky, T., Kapinga, I., Mshana, G., et al (2020). Couples data from north-western Tanzania: Insights from a survey of male partners of women enrolled in the MAISHA cluster randomized trial of an intimate partner violence prevention intervention. *PLoS ONE*, *15*(10).	Risk factors for IPV using couples data.	Physical, sexual	Partner	Individual/ relational	Women n= 1,940 Men n= 452
	Abramsky, T., Lees, S., Stöckl, H., et al. (2019). Women's income and risk of intimate partner violence: Secondary findings from the MAISHA cluster randomised trial in North-Western Tanzania. *BMC Public Health*, *19*(1).	Women's income and IPV experience.	Physical, sexual, economic	Partner	Individual/ relational	
	Kapiga, S., Harvey, S., Muhammad, A. K., Stöckl, H., Mshana, G., Hashim, R., Hansen, C., Lees, S., & Watts, C. (2017). Prevalence of intimate partner violence and abuse and	Prevalence and risk factors for IPV among women.	Physical, sexual, psychological,	Partner	Individual/ relational	

Mannell J, *et al. BMJ Global Health* 2022; 7:e007704. doi: 10.1136/bmjgh-2021-007704

Supplementary material:
Risk factors for violence against women in high-prevalence settings: A mixed methods systematic review and meta-synthesis

Study name/ design	Reference	Focus	VAW type(s)	Perpetrator(s)	Highest level of analysis	Sample
	associated factors among women enrolled into a cluster randomised trial in northwestern Tanzania. *BMC Public Health*, *17*(1).		economic, controlling behaviours			
**MAP project (Men as Partners)** Quantitative; cross-sectional	Kazaura, M. R., Ezekiel, M. J., & Chitama, D. (2016). Magnitude and factors associated with intimate partner violence in mainland Tanzania. *BMC Public Health*, *16*(1).	Risk factors for IPV experience among women.	Physical, sexual, psychological	Partner	Individual/ relational	Women n= 471
**Sauti project 2015** Quantitative; cross-sectional	Nyato, D., Materu, J., Kuringe, E., et al. (2019). Prevalence and correlates of partner violence among adolescent girls and young women: Evidence from baseline data of a cluster randomised trial in Tanzania. *PLoS ONE*, *14*(10)	Prevalence and risk factors for IPV among adolescent girls and young women.	Physical, sexual, psychological	Partner	Individual/ relational	Women n= 2,276
**Shikamana Project 2015 – 2016** Quantitative; cross-sectional	Hendrickson, Z. M., Leddy, A. M., Galai, N., et al. (2018). Work-related mobility and experiences of gender-based violence among female sex workers in Iringa, Tanzania: A cross-sectional analysis of baseline data from Project Shikamana. *BMJ Open*, *8*(9).	Work-related mobility and violence among female sex-workers.	Physical, sexual	Partner, non-partner	Individual/ relational	Women n= 496
**Vijana Vijiweni II 2013 – 2014** Quantitative; cross-sectional	Mulawa, M. I., Reyes, H., Foshee, et al. (2018). Associations Between Peer Network Gender Norms and the Perpetration of Intimate Partner Violence Among Urban Tanzanian Men: a Multilevel Analysis. *Prevention Science*, *19*(4), 427–436.	Peer network gender norms and IPV perpetration among urban men.	Physical	Partner	Individual/ relational	Men n= 1,113
	Mulawa, M., Kajula, L. J., Yamanis, T. et al. (2018). Perpetration and Victimization of Intimate Partner Violence Among Young Men and Women in Dar es Salaam, Tanzania. *Journal of Interpersonal Violence*, *33*(16), 2486–2511.	Risk factors for IPV experience and perpetration among young men and women.	Physical, sexual, psychological	Partner	Community	
**Vijana Vijiweni II 2013 – 2014** Qualitative; in-depth interviews	Mulawa, M. I., Kajula, L. J., & Maman, S. (2018). Peer network influence on intimate partner violence perpetration among urban Tanzanian men. *Culture, Health and Sexuality*, *20*(4), 474–488.	Peer network influence on IPV perpetration among urban men.	Physical	Partner		Men n= 40
**WHO Multi-Country Study on Women's Health and Domestic Violence against Women 2000 – 2004** Quantitative; cross-sectional	Vyas, S., Jansen, H. A. F. M., Heise, L., & Mbwambo, J. (2015). Exploring the association between women's access to economic resources and intimate partner violence in Dar es Salaam and Mbeya, Tanzania. *Social Science and Medicine*, *146*, 307–315.	Women's access to economic resources and IPV experience.	Physical, sexual	Partner	Community	Women n= 3,270
	Stöckl, H., Watts, C., & Kilonzo Mbwambo, J. K. (2010). Physical violence by a partner during pregnancy in Tanzania: Prevalence and risk factors. *Reproductive Health Matters*, *18*(36), 171–180.	Prevalence and risk factors for physical IPV during pregnancy.	Physical	Partner	Individual/ relational	

Supplementary material:
Risk factors for violence against women in high-prevalence settings: A mixed methods systematic review and meta-synthesis

Study name/ design	Reference	Focus	VAW type(s)	Perpetrator(s)	Highest level of analysis	Sample
Quantitative; cross-sectional	Aloyce, Z., Larson, E., Komba, A., Mwimba, A., Kaale, A., Minja, A., Siril, H., Kamala, J., Somba, M., Ngakongwa, F., Kaaya, S., & Fawzi, M. C. S. (2020). Prevalence and factors associated with intimate partner violence after HIV status disclosure among pregnant women with depression in Tanzania. *AIDS Care*, 1–7.	Prevalence and risk factors for IPV after HIV disclosure among pregnant women.	Physical, sexual, psychological	Partner	Individual/ relational	Women n= 659
	Messersmith, L. J., Halim, N., Steven Mzilangwe, E., et al. (2017). Childhood Trauma, Gender Inequitable Attitudes, Alcohol Use and Multiple Sexual Partners: Correlates of Intimate Partner Violence in Northern Tanzania. *Journal of Interpersonal Violence*, online.	Childhood trauma, gender inequitable attitudes, alcohol use, multiple partners and IPV among women.	Physical, sexual, psychological, economic	Partner	Individual/ relational	Women n= 450<br>Men n= 450
	Sigalla, G. N., Rasch, V., Gammeltoft, T., et al. (2017). Social support and intimate partner violence during pregnancy among women attending antenatal care in Moshi Municipality, Northern Tanzania. *BMC Public Health*, *17*(1).	Social support and IPV during pregnancy among women attending antenatal care.	Physical, sexual, psychological	Partner	Individual/ relational	Women n= 1,116
	Laisser, R., Nyström, L., & Emmelin, M. (2014). Health-care workers' attitudes and perceptions of intimate partner violence against women in Tanzania. *African Journal of Midwifery and Women's Health*, *8*(1), 28–35.	Healthcare workers' perceptions of IPV.	Physical, sexual, psychological	Partner	Individual/ relational	Men n= 75
	Prabhu, M., Mchome, B., Ostermann, J., et al. (2011). Prevalence and correlates of intimate partner violence among women attending HIV voluntary counseling and testing in northern Tanzania, 2005-2008. *International Journal of Gynecology & Obstetrics*, *113*(1), 63–67.	Prevalence and risk factors for IPV among women attending HIV testing and counselling.	Physical, sexual, psychological	Partner	Individual/ relational	Women n= 2,436
	McCloskey, L. A., Williams, C., & Larsen, U. (2005). Gender inequality and intimate partner violence among women in Moshi, Tanzania. *International Family Planning Perspectives*, *31*(3), 124–130.	Gender inequality and IPV experience among women.	Physical, sexual	Partner	Individual/ relational	Women n= 1,444
Qualitative; in-depth interviews	Vyas, S., Mbwambo, J., & Heise, L. (2015). Women's Paid Work and Intimate Partner Violence: Insights from Tanzania. *Feminist Economics*, *21*(1), 35–58.	Women's perspectives on paid work and IPV.	Physical, sexual, economic	Partner		Women n= 20
	Lary, H., Maman, S., Katebalila, M., & Mbwambo, J. (2004). Exploring the Association between HIV and Violence: Young People's Experiences with Infidelity, Violence and Forced Sex in Dar es Salaam, Tanzania. *Family Planning Perspectives*, *30*(4), 200–206.	Young people's experiences with infidelity, violence and forced sex.	Physical, sexual	Partner, family member		Women n= 20<br>Men n= 40
Qualitative; focus group discussions	Abeid, M., Muganyizi, P., Olsson, P., et al. (2014). Community perceptions of rape and child sexual abuse: a qualitative study	Community perceptions of rape	Sexual	Partner, family member,		Community members n=

Supplementary material:
Risk factors for violence against women in high-prevalence settings: A mixed methods systematic review and meta-synthesis

Study name/ design	Reference	Focus	VAW type(s)	Perpetrator(s)	Highest level of analysis	Sample
	in rural Tanzania. *BMC International Health and Human Rights*, *14*(1), 23.	and child sexual abuse.		stranger		54
	Laisser, R. M., Nyström, L., Lugina, H. I., & Emmelin, M. (2011). Community perceptions of intimate partner violence - a qualitative study from urban Tanzania. *BMC Women's Health*, *11*(1), 13.	Community perceptions of intimate partner violence.	Physical, sexual	Partner		Key informants n = 75
Qualitative; combination of methods	Manji, K., Heise, L., & Cislaghi, B. (2020). Couples' Economic Equilibrium, Gender Norms and Intimate Partner Violence in Kirumba, Tanzania. *Violence against Women*, *26*(15–16), 2062–2082.	Couples' economic equilibrium, gender norms and intimate partner violence.	Physical, economic	Partner		Women n= 58<br>Men n= 58
Qualitative; ethnographic	Kenny, E. (2019). Narrating Rape: An Ethnographic Case-Study of Non-Consensual Sexual Debut in Tanzania. *Journal of Aggression, Maltreatment and Trauma*, *28*(1), 106–125.	An ethnographic case-study of non-consensual sexual debut in Tanzania.	Sexual	Stranger		Women n= 1
Mixed methods	Rehema, M., & Prisca, K. (2016). The Causes of Intimate Partner Violence in Babati District - University College London. *International Journal of Innovation and Applied Studies*, *17*(4).	Causes of IPV.	Physical, sexual, economic	Partner		Women n= 50<br>Men n= 20<br>Key informants n= 24
	Grabe, S., Grose, R. G., & Dutt, A. (2015). Women's Land Ownership and Relationship Power: A Mixed Methods Approach to Understanding Structural Inequities and Violence Against Women. *Psychology of Women Quarterly*, *39*(1), 7–19.	Women's land ownership, relationship power and violence against women.	Physical, psychological	Partner		Women n= 225
**Timor-Leste**						
**DHS 2016**<br>Quantitative; cross-sectional	Pengpid, S. (2018). Intimate partner victimization and perpetration among female adolescents and adults in Timor-Leste. *Gender & Behaviour*, *16*(1), 11055–11064.	Risk factors for IPV among female adolescents and women.	Physical, sexual	Partner	Individual/ relational	Women n= 3,694
**DILI birth cohort study data 2013 – 2015**<br>Quantitative; cross-sectional	Rees, S., Mohsin, M., Tay, et al. (2018). Risk of perpetrating intimate partner violence amongst men exposed to torture in conflict-affected Timor-Leste. *Global Mental Health*, *5*, e23.	Exposure to torture during conflict and IPV perpetration among men.	Physical, sexual, psychological	Partner	Individual/ relational	Women n= 1,672<br>Men n= 889
	Rees, S., Mohsin, M., Tay, A. K., et al. (2017). Associations between bride price stress and intimate partner violence amongst pregnant women in Timor-Leste. *Globalization and Health*, *13*(1).	Bride price stress and IPV among pregnant women.	Physical, psychological	Partner	Individual/ relational	
Qualitative; combination of methods	Rees, S., Thorpe, R., Tol, W., et al. (2015). Testing a cycle of family violence model in conflict-affected, low-income countries: A qualitative study from Timor-Leste. *Social Science*	Intergenerational transmission of violence within	Physical	Partner		Women n= 77

Supplementary material:
Risk factors for violence against women in high-prevalence settings: A mixed methods systematic review and meta-synthesis

Study name/ design	Reference	Focus	VAW type(s)	Perpetrator(s)	Highest level of analysis	Sample
	and Medicine, *130*, 284–291.	families.				
**Uganda**						
**DHS 2006** Quantitative; cross-sectional	Ogland, E. G., Xu, X., Bartkowski, J. P., & Ogland, C. P. (2014). Intimate Partner Violence Against Married Women in Uganda. *Journal of Family Violence*, *29*(8), 869–879.	Risk factors for married women's experience of IPV.	Physical, sexual, psychological	Partner	Individual/ relational	Women n= 2,087
	Tumwesigye, N. M., Kyomuhendo, G. B., Greenfield, T. K., & Wanyenze, R. K. (2012). Problem drinking and physical intimate partner violence against women: evidence from a national survey in Uganda. *BMC Public Health*, *12*(1), 399.	Men's alcohol use and women's experience of IPV.	Physical	Partner	Individual/ relational	
	Speizer, I. S. (2010). Intimate partner violence attitudes and experience among women and men in Uganda. *Journal of Interpersonal Violence*, *25*(7), 1224–1241.	IPV attitudes and experience among men and women.	Physical, sexual	Partner	Individual/ relational	
**DHS 2011** Quantitative; cross-sectional	Kadengye, D. T., Iddi, S., Hunter, L., & McCoy, S. I. (2019). Effectiveness of Potential Interventions to Change Gendered Social Norms on Prevalence of Intimate Partner Violence in Uganda: a Causal Inference Approach. *Prevention Science*, *20*(7), 1043–1053.	Causal inference approach to changing gendered social norms and IPV.	Physical, sexual, psychological	Partner	Community	Women n = 2,056
	Zegenhagen, S., Ranganathan, M., & Buller, A. M. (2019). Household decision-making and its association with intimate partner violence: Examining differences in men's and women's perceptions in Uganda. *SSM - Population Health*, *8*, 100442.	Men's and women's household decision-making and IPV experience.	Physical	Partner	Individual/ relational	
	Odimegwu, C., & Frade, S. (2018). The influence of adolescent age at first union on physical intimate partner violence and fertility in Uganda: A path analysis. *SAJCH South African Journal of Child Health*, *12*(Special Issue), S51–S56.	Age at first union, IPV experience and fertility among women.	Physical	Partner	Individual/ relational	
	Wandera, S. O., Kwagala, B., Ndugga, P., & Kabagenyi, A. (2015). Partners' controlling behaviors and intimate partner sexual violence among married women in Uganda Global health. *BMC Public Health*, *15*(1).	Partner's controlling behaviour and women's experience of sexual IPV.	Sexual	Partner	Individual/ relational	
	Kwagala, B., Wandera, S. O., Ndugga, P., & Kabagenyi, A. (2013). Empowerment, partner's behaviours and intimate partner physical violence among married women in Uganda. *BMC Public Health*, *13*(1).	Empowerment, partner's behaviour and women's IPV experience.	Physical	Partner	Individual/ relational	
**DHS 2016** Quantitative; cross-sectional	Gubi, D., Nansubuga, E., & Wandera, S. O. (2020). Correlates of intimate partner violence among married women in Uganda: a cross-sectional survey. *BMC Public Health*, *20*(1).	Risk factors for IPV experience among women.	Physical, sexual, psychological	Partner	Individual/ relational	Women n= 9,319
	Amegbor, P. M., & Pascoe, L. (2019). Variations in Emotional, Sexual, and Physical Intimate Partner Violence Among Women in Uganda: A Multilevel Analysis. *Journal of*	National variations in risk factors for IPV.	Physical, sexual, psychological	Partner	Regional	

Supplementary material:
Risk factors for violence against women in high-prevalence settings: A mixed methods systematic review and meta-synthesis

Study name/ design	Reference	Focus	VAW type(s)	Perpetrator(s)	Highest level of analysis	Sample
	Interpersonal Violence. Online.					
	Amegbor, P. M., & Rosenberg, M. W. (2019). What geography can tell us? Effect of higher education on intimate partner violence against women in Uganda. *Applied Geography*, *106*, 71–81.	Geographical variation in effect of higher education on IPV.	Physical, sexual, psychological	Partner	District	
**DHS 2006 and 2011** Quantitative; cross-sectional	Valentine, A., Akobirshoev, I., & Mitra, M. (2019). Intimate Partner Violence among Women with Disabilities in Uganda. *International Journal of Environmental Research and Public Health*, *16*(6), 947.	Disability and risk of IPV among women.	Physical, sexual, psychological	Partner	Individual/ relational	Women n= 8,592
**CRANE survey 2012** Quantitative; cross-sectional)	Schwitters, A., Swaminathan, M., Serwadda, D.,et al. (2015). Prevalence of Rape and Client-Initiated Gender-Based Violence Among Female Sex Workers: Kampala, Uganda, 2012. *AIDS and Behavior*, *19*(1), 68–76.	Prevalence of rape and client-initiated violence among female sex workers.	Sexual, psychological, economic	Client	Individual/ relational	Women n= 1,467
**Essential Child Health and Nutrition Project in Uganda** Quantitative; cross-sectional	Karamagi, C. A. S., Tumwine, J. K., Tylleskar, T., & Heggenhougen, K. (2006). Intimate partner violence against women in eastern Uganda: Implications for HIV prevention. *BMC Public Health*, *6*.	Risk factors for IPV among women.	Physical, sexual	Partner	Individual/ relational	Women n= 457
**Gulu Sexual Health Project 2012** Quantitative; cross-sectional	Erickson, M., Goldenberg, S. M., Muzaaya, G., et al. (2017). Interpersonal and structural contexts of intimate partner violence among female sex workers in conflict-affected northern Uganda. *Women & Health.* 58(7), 759-773.	Risk factors for IPV among female sex workers.	Physical, sexual	Partner	Individual/ relational	Women n= 379
**Rakai Community Open Cohort Study data 2000 – 2018** Quantitative; cross-sectional	Zablotska, I. B., Gray, R. H., Koenig, M. A., et al. (2009). Alcohol use, intimate partner violence, sexual coercion and HIV among women aged 15-24 in Rakai, Uganda. *AIDS and Behavior*, *13*(2), 225–233.	Alcohol use, IPV, sexual coercion and HIV among women.	Physical, sexual	Partner	Individual/ relational	Women n= 15,081 Men n= 6,531
	Kouyoumdjian, F. G., Calzavara, L. M., Bondy, et al. (2013). Risk factors for intimate partner violence in women in the Rakai Community Cohort Study, Uganda, from 2000 to 2009. *BMC Public Health*, *13*(1), 566.	Risk factors for IPV experience among women.	Physical, sexual, psychological	Partner	Individual/ relational	
	Sabri, B., Wirtz, A. L., Ssekasanvu, J., et al. (2019). Intimate partner violence, HIV and sexually transmitted infections in fishing, trading and agrarian communities in Rakai, Uganda. *BMC Public Health*, *19*(1), 1–16.	IPV, HIV and sexually transmitted infections among men and women.	Physical, sexual	Partner	Individual/ relational	
	Miller, A. P., Pitpitan, E. v., Nabukalu, D., et al. (2020). Transactional Sex, Alcohol Use and Intimate Partner Violence Against Women in the Rakai Region of Uganda. *AIDS and*	Transactional sex, alcohol use and IPV experience among	Physical, sexual, psychological	Partner	Individual/ relational	

Supplementary material:
Risk factors for violence against women in high-prevalence settings: A mixed methods systematic review and meta-synthesis

Study name/ design	Reference	Focus	VAW type(s)	Perpetrator(s)	Highest level of analysis	Sample
	Behavior.	women.				
**SASA! Trial 2007 – 2012** Quantitative; cross-sectional	Abramsky, T., Devries, K. M., Michau, L.et al. (2016). Ecological pathways to prevention: How does the SASA! community mobilisation model work to prevent physical intimate partner violence against women? *BMC Public Health*, *16*(1), 339.	Community mobilisation intervention to prevent violence against women.	Physical, sexual	Partner	Community	Women n= 4,385
	Abramsky, T., Devries, K., Kiss, L., et al. (2014). Findings from the SASA! Study: a cluster randomized controlled trial to assess the impact of a community mobilization intervention to prevent violence against women and reduce HIV risk in Kampala, Uganda. *BMC Medicine*, *12*(1), 122.	Community mobilisation intervention to prevent violence against women.	Physical	Partner	Community	
	Francisco, L. v. (2010). The synergy between intimate partner violence and HIV: baseline findings from the SASA! study, a cluster randomised controlled community trial in Kampala, Uganda. Thesis submitted to Johns Hopkins University, Baltimore, USA.	Risk factors for IPV and HIV.	Physical, sexual, psychological	Partner	Individual/ relational	
**SASA! Trial 2007 – 2012** Qualitative; in-depth interviews	Kyegombe, N., Starmann, E., Devries, K. M., et al. (2014). “SASA! is the medicine that treats violence”. Qualitative findings on how a community mobilisation intervention to prevent violence against women created change in Kampala, Uganda. *Global Health Action*, *7*(1).	How a community mobilisation intervention to prevent violence against women created change.	Physical, sexual, economic	Partner		Women n= 20 Men n= 20
**Survey of War Affected Youth** Qualitative; in-depth interviews	Annan, J., & Brier, M. (2010). The risk of return: Intimate partner violence in Northern Uganda’s armed conflict. *Social Science and Medicine*, *70*(1), 152–159.	IPV during Northern Uganda’s armed conflict.	Physical, sexual, psychological	Partner		Women n= 21
**Uganda AIDS Rural Treatment Outcomes (UARTO) cohort 2011 – 2015** Quantitative; cross-sectional	Young, C. R., Kaida, A., Kabakyenga, J., et al. (2018). Prevalence and correlates of physical and sexual intimate partner violence among women living with HIV in Uganda. *PLoS ONE*, *13*(8).	Prevalence and risk factors for IPV among HIV positive women.	Physical, sexual	Partner	Individual/ relational	Women n= 455
	Conroy, A. A., Tsai, A. C., Clark, G. et al (2016). Relationship Power and Sexual Violence Among HIV-Positive Women in Rural Uganda. *AIDS and Behavior*, *20*(9), 2045–2053.	Relationship and IPV among HIV positive women.	Sexual	Partner	Individual/ relational	
**Uganda national survey of HIV-infected women in care 2016** Quantitative;	Kabwama, S. N., Bukenya, J., Matovu, J. K. B. et al. (2019). Intimate partner violence among HIV positive women in care - Results from a national survey, Uganda 2016. *BMC Women’s Health*, *19*(1).	Risk factors for IPV among HIV positive women in care.	Physical, sexual	Partner	Individual/ relational	Women n= 5,198

Supplementary material:
Risk factors for violence against women in high-prevalence settings: A mixed methods systematic review and meta-synthesis

Study name/ design	Reference	Focus	VAW type(s)	Perpetrator(s)	Highest level of analysis	Sample
cross-sectional						
Quantitative; cross-sectional	Black, E., Worth, H., Clarke, S., et al. (2019). Prevalence and correlates of intimate partner violence against women in conflict affected northern Uganda: A cross-sectional study. *Conflict and Health*, *13*(1).	Prevalence and risk factors for IPV among women affected by conflict.	Physical, sexual, psychological	Partner	Individual/ relational	Women n= 409
	Epuitai, J., Udho, S., Auma, A. G., & Nabirye, R. C. (2019). Intimate partner violence among pregnant women in Uganda. *African Journal of Midwifery and Women's Health*, *13*(2), 1–5.	Risk factors for IPV among pregnant women.	Physical, sexual, psychological	Partner	Individual/ relational	Women n= 181
	Logie, C. H., Okumu, M., Mwima, S., et al. (2019). Social ecological factors associated with experiencing violence among urban refugee and displaced adolescent girls and young women in informal settlements in Kampala, Uganda: A cross-sectional study. *Conflict and Health*, *13*(1), 60.	Risk factors for violence among urban refugee and displaced adolescent girls and young women.	Physical, sexual, psychological	Partner	Community	Women n= 333
	Mootz, J. J., Muhanguzi, F. K., Panko, P., Mangen, P. O., Wainberg, M. L., Pinsky, I., & Khoshnood, K. (2018). Armed conflict, alcohol misuse, decision-making, and intimate partner violence among women in Northeastern Uganda: a population level study. *Conflict and Health*, *12*(1), 37.	Armed conflict, alcohol misuse, decision-making and IPV among women.	Physical, psychological	Partner	Individual/ relational	Women n= 605
	Kinyanda, E., Weiss, H. A., Mungherera, Met al. (2016). Intimate partner violence as seen in post-conflict eastern Uganda: prevalence, risk factors and mental health consequences. *BMC International Health and Human Rights*, *16*(1), 5.	Risk factors for IPV among women.	Physical, sexual, psychological	Partner	Individual/ relational	Women n= 1,110
	Lundberg, P., Nakasujja, N., Musisi, S., et al. (2015). Sexual risk behavior, sexual violence, and HIV in persons with severe mental illness in uganda: Hospital-based cross-sectional study and national comparison data. *American Journal of Public Health*, *105*(6), 1142–1148.	Sexual risk behaviour, sexual violence and HIV among people with mental illness.	Sexual	Partner, stranger	Individual/ relational	Women n= 602
	Anyango, J. F., & Muliira, J. K. (2009). Intimate partner violence among pregnant women in rural Uganda. *African Journal of Midwifery and Women's Health*, *3*(4), 187–192.	Risk factors for IPV among pregnant women.	Physical, sexual, psychological	Partner	Individual/ relational	Women n= 120
	Saile, R., Neuner, F., Ertl, V., & Catani, C. (2013). Prevalence and predictors of partner violence against women in the aftermath of war: A survey among couples in Northern Uganda. *Social Science and Medicine*, *86*, 17–25.	Prevalence and risk factors for IPV among women.	Physical, sexual, psychological, controlling behaviours	Partner	Individual/ relational	Women n= 235<br>Men n= 235
	Shuaib, F. M. B., Ehiri, J. E., Jolly, P., Zhang, Q., Emusu, D., Ngu, J., Foushee, H., Katongole, D., Kirby, R., & Wabwire-	Risk factors for IPV among women in HIV	Sexual	Partner	Individual/ relational	Women n= 250

Supplementary material:
Risk factors for violence against women in high-prevalence settings: A mixed methods systematic review and meta-synthesis

Study name/ design	Reference	Focus	VAW type(s)	Perpetrator(s)	Highest level of analysis	Sample
	Mangen, F. (2012). Sexual violence and associated factors among women in HIV discordant and concordant relationships in Uganda. *International Journal of Adolescent Medicine and Health*, *24*(2), 125–133.	concordant and discordant unions.				
	Agardh, A., Tumwine, G., Asamoah, B. O., & Cantor-Graae, E. (2012). The Invisible Suffering: Sexual Coercion, Interpersonal Violence, and Mental Health - A Cross-Sectional Study among University Students in South-Western Uganda. *PLoS ONE*, *7*(12).	Risk factors for sexual violence among female university students.	Sexual	Partner, stranger	Individual/ relational	Women n= 633
	Osinde, M. O., Kaye, D. K., & Kakaire, O. (2011). Intimate partner violence among women with HIV infection in rural Uganda: Critical implications for policy and practice. *BMC Women's Health*, *11*(1), 50.	Risk factors for IPV among HIV positive women.	Physical, sexual, psychological	Partner	Individual/ relational	Women n= 317
	Maclachlan, E., Neema, S., Luyirika, E., et al. (2009). Women, economic hardship and the path of survival: HIV/AIDS risk behavior among women receiving HIV/AIDS treatment in Uganda. *AIDS Care*, *21*(3), 355–367.	Economic hardship, HIV and IPV among women receiving HIV treatment.	Sexual	Partner, stranger	Individual/ relational	Women n= 377
	Koenig, M. A., Lutalo, T., Zhao, F., et al. (2003). Domestic violence in rural Uganda: Evidence from a community-based study. *Bulletin of the World Health Organization*, *81*(1), 53–60.	Risk factors for IPV among women.	Physical, psychological	Partner	Individual/ relational	Women n= 5,109
Quantitative; prospective cohort	Emusu, D. (2007). *Sexual Violence and Correlates Among Women in HIV Discordant Union*. University of Alabama at Birmingham.	Risk factors for sexual violence among women in HIV discordant unions.	Sexual	Partner	Individual/ relational	Women n= 250
Qualitative; in-depth interviews	Lundberg, P., Johansson, E., Okello, E., et al. (2012). Sexual risk behaviours and sexual abuse in persons with severe mental illness in Uganda: A qualitative study. *PLoS ONE*, *7*(1).	Sexual risk behaviour and abuse in people with mental illness.	Sexual	Partner, stranger		Men n= 20
	Hague, G., Thiara, R. K., & Turner, A. (2011). Bride-price and its links to domestic violence and poverty in Uganda: A participatory action research study. *Women's Studies International Forum*, *34*(6), 550–561.	Bride-price and its links to domestic violence and poverty.	Physical, psychological, sexual, economic	Partner, family member		Women n= 170<br>Men n= 57<br>Key informants n = 27
	Emusu, D., Ivankova, N., Jolly, P., et al. (2009). Experience of sexual violence among women in HIV discordant unions after voluntary HIV counselling and testing: a qualitative critical incident study in Uganda. *AIDS Care*, *21*(11), 1363–1370.	Sexual violence among women in HIV discordant unions.	Sexual	Partner		Women n= 26
Qualitative; focus group discussions	Ager, A., Bancroft, C., Berger, E., & Stark, L. (2018). Local constructions of gender-based violence amongst IDPs in northern Uganda: analysis of archival data collected using a	Constructions of gender-based violence among	Physical, sexual	Partner, stranger		FGDs n= 64 (breakdown of participants

Supplementary material:
Risk factors for violence against women in high-prevalence settings: A mixed methods systematic review and meta-synthesis

Study name/ design	Reference	Focus	VAW type(s)	Perpetrator(s)	Highest level of analysis	Sample
	gender- and age-segmented participatory ranking methodology. *Conflict and Health*, *12*(1), 10.	internally displaced persons.				not available)
	Mullinax, M., Higgins, J., Wagman, J., et al. (2013). Community understandings of and responses to gender equality and empowerment in Rakai, Uganda. *Global Public Health*, *8*(4), 465–478.	Community understandings of and responses to gender equality and empowerment.	Not specified	Partner		Women n= 48<br>Men n= 46
	Cash, K. (2011). What's shame got to do with it: forced sex among married or steady partners in Uganda. *African Journal of Reproductive Health*, *15*(3), 25–40.	Forced sex among married or steady partners in Uganda.	Sexual	Partner		Women n= 202<br>Men n= 248
Qualitative; combination of methods	Kwiringira, J. N., Mutabazi, M. M., Mugumya, F., et al. (2018). Experiences of Gender Based Violence among Refugee Populations in Uganda: Evidence from Four Refugee Camps. *Eastern Africa Social Science Research Review*, *34*(1), 291–311.	Experiences of gender-based violence among refugee populations.	Physical, sexual, psychological, economic	Partner, family member, stranger		Women n= 16<br>FGDs with men and women n= 20 (breakdown not available)
	Mootz, J. J., Stabb, S. D., & Mollen, D. (2017). Gender-Based Violence and Armed Conflict. *Psychology of Women Quarterly*, *41*(3), 368–388.	Gender-based violence and armed conflict.	Physical, sexual, psychological, economic	Partner, family members		Women n= 34<br>Men n = 43
	Namy, S., Carlson, C., O'Hara, K., et al. (2017). Towards a feminist understanding of intersecting violence against women and children in the family. *Social Science and Medicine*, *184*, 40–48.	Feminist understanding of intersecting violence against women and children in the family.	Physical	Partner		Women n= 28<br>Men n= 27
	Kaye, D. K., Mirembe, F., Ekstrom, A., et al. (2005). Implications of bride price on domestic violence and reproductive health in Wakiso District, Uganda. *African Health Sciences*, *5*(4), 300–303.	Bride price and domestic violence in Wakiso district, Uganda.	Physical, sexual	Partner		FGDs n= 10 (breakdown not available)<br>Key informants n= 12
	Kaye, D. K., Mirembe, F., Ekstrom, A. et al. (2005). The social construction and context of domestic violence in Wakiso District, Uganda. *Culture, Health and Sexuality*, *7*(6), 625–635.	The social construction and context of domestic violence in Wakiso district, Uganda.	Physical, sexual, psychological	Partner		FGDs n= 10 (breakdown not available)
**Vanuatu**						
**Vanuatu National Survey on Women's Lives**	Vanuatu National Statistics Office. (2011). *Vanuatu National Survey on Women's Lives and Family Relationships*. Vanuatu Women's Centre. Port Villa, Vanuatu.	Risk factors for IPV experience among women.	Physical, sexual	Partner	Individual/ relational	Women n= 2,337

Supplementary material:
Risk factors for violence against women in high-prevalence settings: A mixed methods systematic review and meta-synthesis

Study name/ design	Reference	Focus	VAW type(s)	Perpetrator(s)	Highest level of analysis	Sample
**and Family Relationships** Quantitative; cross-sectional						
Quantitative; cross-sectional	McKelvie, S., Leodoro, B., Sala, T., Tran, T., & Fisher, J. (2020). Prevalence, Patterns, and Determinants of Intimate Partner Violence Experienced by Women Who Are Pregnant in Sanma Province, Vanuatu. *Journal of Interpersonal Violence*, online.	Prevalence and risk factors for IPV among pregnant women.	Physical, sexual, psychological, controlling behaviours	Partner	Individual/ relational	Women n= 192
**Zambia**						
**DHS 2001** Quantitative; cross-sectional	Okenwa, L., & Lawoko, S. (2010). Social indicators and physical abuse of women by intimate partners: A study of women in Zambia. *Violence and Victims*, *25*(2), 278–288.	Risk factors for IPV among women.	Physical	Partner	Individual/ relational	Women n= 5,029
	Klomegah, R. Y. (2008). Intimate Partner Violence (IPV) in Zambia: An Examination of Risk Factors and Gender Perceptions. *Journal of Comparative Family Studies*, *39*(4), 557–569.	Risk factors for IPV among women.	Physical	Partner	Individual/ relational	
**DHS 2007** Quantitative; cross-sectional	Shinabarger Reed, C. K., (2010). *Intimate Partner Violence and Infertility in Zambia.* Texas Woman's University, Denton, Texas.	Infertility and IPV among women.	Physical, sexual, psychological	Partner	Individual/ relational	Women n= 5,029
**Zambia Emory HIV Research Project 2012 – 2015** Quantitative; cross-sectional	Malama, K., Sagaon-Teyssier, L., Parker, R., (2019). Client-Initiated Violence Against Zambian Female Sex Workers: Prevalence and Associations With Behavior, Environment, and Sexual History. *Journal of Interpersonal Violence*. Online.	Prevalence and risk factors of client-initiated violence among female sex-workers.	Physical	Clients	Individual/ relational	Women n= 419
**Maternity waiting home intervention 2016** (quantitative; cross-sectional)	Munro-Kramer, M. L., Scott, N., Boyd, C. J., Veliz, P. T., Murray, S. M., Musonda, G., & Lori, J. R. (2018). Postpartum physical intimate partner violence among women in rural Zambia. *International Journal of Gynecology and Obstetrics*, *143*(2), 199–204.	Risk factors for IPV experience among postpartum women.	Physical	Partner	Individual/ relational	Women n= 2,381
**Stamping Out and Preventing Gender Based Violence in Zambia (STOP GBV)]** Qualitative; in-	Samuels, F., Ndubani, P., Walker, D., & Simbaya, J. (2015). *Baseline Study: Stamping Out and Preventing Gender Based Violence (STOP GBV) in Zambia*. Overseas Development Institute. London, UK.	Preventing gender-based violence.	Physical, psychological, economic	Partner, family member		Women n= 116 Men n= 109

Supplementary material:
Risk factors for violence against women in high-prevalence settings: A mixed methods systematic review and meta-synthesis

Study name/ design	Reference	Focus	VAW type(s)	Perpetrator(s)	Highest level of analysis	Sample
depth interviews, FGDs						
**Violence and Alcohol Treatment Study Trial** Quantitative; cross-sectional	Fine, S. L., Kane, J. C., Murray, S. M., et al. (2019). The Role of Violence Acceptance and Inequitable Gender Norms in Intimate Partner Violence Severity Among Couples in Zambia. *Journal of Interpersonal Violence*. Online.	Violence acceptance, gender norms and IPV severity among couples.	Physical, sexual	Partner		Women n= 247<br>Men n= 247
Quantitative; cross-sectional	Birbeck, G., Chomba, E., Atadzhanov, et al. (2007). The social and economic impact of epilepsy in Zambia: a cross-sectional study. *The Lancet Neurology*, 39.	Epilepsy and risk of IPV among women.	Physical, sexual	Partner, family member	Individual/ relational	Women n= 338
Qualitative; in-depth interviews	Sichimba, F., Cindy Nakazwe, K., & Phiri, T. (2020). Untold Stories of Women Living in Violence: Lived Realities of Why Women Stay: A Case Study of Ngombe and Kanyama Compounds in Lusaka. *Journal of Aggression, Maltreatment & Trauma*, *29*(7), 767–784.	Women's experiences of staying in violent relationships.	Physical, psychological	Partner		Women n= 25
	Murray, L. K., Haworth, A., Semrau, K., et al. (2006). Violence and abuse among HIV-infected women and their children in Zambia: A qualitative study. *Journal of Nervous and Mental Disease*, *194*(8), 610–615.	Violence and abuse among HIV-infected women and their children.	Physical	Partner		Key informants n= 22
Qualitative; combination of methods	Murray, S. M., Skavenski Van Wyk, S., et al. (2021). A qualitative exploration of mechanisms of intimate partner violence reduction for zambian couples receiving the Common Elements Treatment Approach (CETA) intervention. *Social Science and Medicine*, *268*.	Mechanisms of IPV reduction for couples receiving the CETA intervention.	Physical, sexual, psychological	Partner		Women n= 50<br>Men n= 50
**Multi-country studies** (including at least one high-prevalence setting)						
**GENACIS (Gender, Alcohol, and Culture: An International Study) 2000 – 2001** Quantitative; cross-sectional	Bernards, S., & Graham, K. (2013). The Cross-Cultural Association Between Marital Status and Physical Aggression Between Intimate Partners. *Journal of Family Violence*, *28*(4), 403–418.	Marital status and physical IPV among men and women.	Physical	Partner	Individual/ relational	Women n= 657<br>Men n= 612
	Graham, K., Bernards, S., Wilsnack, S. C., & Gmel, G. (2011). Alcohol may not cause partner violence but it seems to make it worse: A cross national comparison of the relationship between alcohol and severity of partner violence. *Journal of Interpersonal Violence*, *26*(8), 1503–1523.	Alcohol use and severity of IPV among men and women.	Physical	Partner	Individual/ relational	
Qualitative; focus-group discussions	Holmila, M., Beccaria, F., Ibanga, A., Graham, K., Hettige, S., Magri, R., Munné, M., Plant, M., Rolando, S., & Tumwesigye, N. (2014). Gender, alcohol and intimate partner violence: Qualitative comparative study. *Drugs: Education, Prevention*	Gender, alcohol and intimate partner violence.	Physical, psychological	Partner		Women n= 7<br>Men n= 6

Supplementary material:
Risk factors for violence against women in high-prevalence settings: A mixed methods systematic review and meta-synthesis

Study name/ design	Reference	Focus	VAW type(s)	Perpetrator(s)	Highest level of analysis	Sample
	and Policy, *21*(5), 398–407.					
**International Men and Gender Equality Survey (IMAGES) 2012** Quantitative; cross-sectional	Flemming, P. J., Mc-Cleary-Sills, J., Morton, et al. (2015). Risk Factors for Men's Lifetime Perpetration of Physical Violence against Intimate Partners: Results from the International Men and Gender Equality Survey (IMAGES) in Eight Countries. *PloS One*, *10*(5).	Risk factors for men's IPV perpetration.	Physical	Partner	Individual/ relational	Men n= 539
**DHS datasets** Quantitative; cross-sectional	Akinyemi, J. O., Somefun, O., Olamijuwon, E. O., de Wet, N., & Odimegwu, C. O. (2020). How long was the honeymoon? Accelerated failure time model of spousal violence onset among women in sub-Saharan Africa. *Women & Health*, *60*(9), 987–999.	Accelerated failure time model of onset of IPV.	Physical	Partner	Individual/ relational	All women<br>Tanzania n=5,795<br>Uganda n= 5,504<br>Zambia n= 5,437<br>Burundi n= 5,839
	Coll, C. V. N., Ewerling, F., García-Moreno, C., Hellwig, F., & Barros, A. J. D. (2020). Intimate partner violence in 46 low-income and middle-income countries: an appraisal of the most vulnerable groups of women using national health surveys. *BMJ Global Health*, *5*(1).	Risk factors for IPV among women in 46 LMICs.	Physical, sexual, psychological	Partner	Individual/relational	All women<br>DRC n=5,691<br>Cameroon n= 4,006<br>Sierra Leone n= 4,315<br>Tanzania n= 7,597<br>Uganda n= 7,536<br>Zambia n= 9,416<br>Angola n= 7,669<br>Afghanistan n= 21,324<br>Timor Leste n= 3,694<br>Gabon n= 4,147<br>Burundi n= 7,366
	Epstein, A., Bendavid, E., Nash, D., Charlebois, E. D., &	Drought and IPV	Physical,	Partner	Societal	All women

Supplementary material:
Risk factors for violence against women in high-prevalence settings: A mixed methods systematic review and meta-synthesis

Study name/ design	Reference	Focus	VAW type(s)	Perpetrator(s)	Highest level of analysis	Sample
	Weiser, S. D. (2020). Drought and intimate partner violence towards women in 19 countries in sub-saharan Africa during 2011-2018: A population-based study. *PLoS Medicine*, *17*(3).	among women in 19 countries in sub-Saharan Africa.	sexual, psychological			Zambia n=7,577 Burundi n=6,364 Cameroon n=3,396 DRC n=4,557 Gabon n=3,112 Sierra Leone n=3,877 Tanzania n=6,452 Uganda n=6,119 Angola n=6,004
	Kiwuwa-Muyingo, S., & Kadengye, D. T. (2020). Prevalence and Risk Factors for Women's Reports of Past-Year Intimate Partner Violence: A Comparative Analysis of Six East African National Surveys. *Journal of Interpersonal Violence*. Online.	Prevalence and risk factors for IPV among women in 6 East African countries.	Physical, sexual, psychological	Partner	Individual/relational	All women Uganda n= 7,536 Burundi n= 7,366 Tanzania n= 7,597
	Wilson, N. (2019). Socio-economic Status, Demographic Characteristics and Intimate Partner Violence. *Journal of International Development*, *31*(7), 632–657.	Socio-economic status, demographic characteristics and IPV among women.	Physical, sexual, psychological	Partner	Individual/relational	Not provided for individual countries included in study
	Behrman, J. A., Peterman, A., & Palermo, T. (2017). Does Keeping Adolescent Girls in School Protect Against Sexual Violence? Quasi-Experimental Evidence From East and Southern Africa. *Journal of Adolescent Health*, *60*(2), 184–190.	Protective effect of girls' education on experience of sexual violence.	Sexual	Partner and stranger	Individual/relational	Women n= 1,028
	Cools, S., & Kotsadam, A. (2017). Resources and Intimate Partner Violence in Sub-Saharan Africa. *World Development*, *95*, 211–230.	Resources and IPV among women in sub-Saharan Africa.	Physical, sexual	Partner	Society	Not provided for individual countries included in study
	Decker, M. R., Latimore, A. D., Yasutake, S., et al. (2015).	Age and IPV among	Physical,	Partner	Individual/r	All women

Supplementary material:
Risk factors for violence against women in high-prevalence settings: A mixed methods systematic review and meta-synthesis

Study name/ design	Reference	Focus	VAW type(s)	Perpetrator(s)	Highest level of analysis	Sample
	Gender-based violence against adolescent and young adult women in low- and middle-income countries. *Journal of Adolescent Health*, *56*(2), 188–196.	adolescent and young adult women in LMICs.	sexual		elational	Tanzania n= 2,249 Uganda n= 723 Zambia n= 1,756 Cameroon n=1,388 DRC n=1,339, Liberia n=1,342 Sao Tome and Principe n=586 Bangladesh n=2,840 Timor-Leste n=640 Bolivia n=3,470
	Bamiwuye, S. O., & Odimegwu, C. (2014). Spousal violence in sub-Saharan Africa: Does household poverty-wealth matter? *Reproductive Health*, *11*(1).	Household poverty-wealth and IPV in sub-Saharan Africa.	Physical, sexual, psychological	Partner	Individual/ relational	All women Cameroon n=3,691 Zambia n=3,010
	Bott, S., Guedes, A., Goodwin, M., & Adams Mendoza, J. (2012). *Violence Against Women in Latin America and the Caribbean: A comparative analysis of population-based data from 12 countries.* Pan American Health Organization. Washington DC, USA.	Risk factors for IPV among women in Latin America and the Caribbean.	Physical, sexual	Partner	Individual/ relational	Not provided for individual countries included in study
	Alio, A. P., Clayton, H. B., Garba, M., et al. (2011). Spousal Concordance in Attitudes Toward Violence and Reported Physical Abuse in African Couples. *Journal of Interpersonal Violence*, *26*(14), 2790–2810.	Spousal concordance in attitudes towards VAW and physical IPV.	Physical	Partner	Individual/ relational	Not provided for individual countries included in study
**Violence against children survey** Quantitative; cross-sectional	Stark, L., Seff, I., Hoover, A., Gordon, R., Ligiero, D., & Massetti, G. (2019). Sex and age effects in past-year experiences of violence amongst adolescents in five countries. *PLoS ONE*, *14*(7).	Age and sex effects on violence among young people.	Physical, sexual	Partner, family member, stranger	Individual/ relational	Women n= 1,968 Men n= 1,771
**WHO Multi-**	Stöckl, H., March, L., Pallitto, C., & Garcia-Moreno, C. (2014).	Prevalence and risk	Physical,	Partner	Individual/	Bangladesh

Supplementary material:
Risk factors for violence against women in high-prevalence settings: A mixed methods systematic review and meta-synthesis

Study name/ design	Reference	Focus	VAW type(s)	Perpetrator(s)	Highest level of analysis	Sample
**Country Study on Women's Health and Domestic Violence against Women 2000 – 2004** Quantitative; cross-sectional	Intimate partner violence among adolescents and young women: Prevalence and associated factors in nine countries: A cross-sectional study. *BMC Public Health*, *14*(1).	factors for IPV among adolescents and young women in 9 countries.	sexual		relational	women n= 3,130<br><br>Tanzania women n= 3,270
	Abramsky, T., Watts, C. H., Garcia-Moreno, et al. (2011). What factors are associated with recent intimate partner violence? findings from the WHO multi-country study on women's health and domestic violence. *BMC Public Health*, *11*(1), 109.	Risk factors for IPV experience among women.	Physical, sexual	Partner	Individual/ relational	
	Garcia-Moreno, C., Jansen, H. A., Ellsberg, M., et al. (2006). Prevalence of intimate partner violence: findings from the WHO multi-country study on women's health and domestic violence. *Lancet*, *368*(9543), 1260–1269.	Prevalence of IPV among women.	Physical, sexual	Partner	Individual/ relational	
Quantitative; cross-sectional	Chirwa, E., Jewkes, R., van der Heijden, I., & Dunkle, K. (2020). Intimate partner violence among women with and without disabilities: a pooled analysis of baseline data from seven violence-prevention programmes. *BMJ Global Health*, *5*(11).	Disability and IPV experience among women.	Physical, psychological, economic	Partner	Individual/ relational	Women n= 933
	Instituto Nacional de Estadística Avenida. (2017). *Encuesta de prevalencia y características de la Violencia contra las mujeres 2016*. La Paz, Bolivia.	Prevalence and risk factors for VAW.	Physical, sexual, psychological, economic	Partner, stranger, manager	Individual/ relational	Not reported for individual countries
	Andersson, N., Ho-Foster, A., Mitchell, et al. (2007). Risk factors for domestic physical violence: national cross-sectional household surveys in eight southern African countries. *BMC Women's Health*, *7*(1), 11.	Risk factors for IPV experience among women.	Physical	Partner	Individual/ relational	Women n= 1,605
Qualitative; in-depth interviews	Atim, L., Mwangoka, M., Martins, L., & Rickard, S. (2020). *Sexual and Gender-Based Violence in the Mining Sector in Africa. Evidence and reflections from the DRC, South Africa, Tanzania & Uganda*. German Federal Ministry for Economic Cooperation and Development (BMZ).	Sexual and Gender-Based Violence in the Mining Sector in Africa.	Physical, psychological, sexual, economic	Partner, stranger		22 key informants
Qualitative; combination of methods	Falb, K., Asghar, K., Pardo, N. M., Hategekimana, J. D. D., Kakay, H., Roth, D., & O'Connor, M. (2020). Developing an Inclusive Conceptual Model for Preventing Violence in the Home in Humanitarian Settings: Qualitative Findings From Myanmar and the Democratic Republic of Congo. *Journal of Interpersonal Violence*. Online.	Conceptual model for preventing violence in the home in humanitarian settings.	Physical, sexual, psychological	Partner, family members		Women n= 75<br>Men n= 73
	Tanabe, M., Nagujjah, Y., Rimal, N., et al. (2015). Intersecting Sexual and Reproductive Health and Disability in Humanitarian Settings: Risks, Needs, and Capacities of Refugees with	Intersecting sexual and reproductive health and disability	Sexual	Stranger		Women n= 15<br>Men n=17

Supplementary material:
Risk factors for violence against women in high-prevalence settings: A mixed methods systematic review and meta-synthesis

Study name/ design	Reference	Focus	VAW type(s)	Perpetrator(s)	Highest level of analysis	Sample
	Disabilities in Kenya, Nepal, and Uganda. *Sexuality and Disability*, *33*(4), 411–427.	in humanitarian settings.				
				**Total:**	Women = 280,360<br>Men = 40,276<br>Key informants = 274	

Supplementary material:
Risk factors for violence against women in high-prevalence settings: A mixed methods systematic review and meta-synthesis

Table 7: Quality assessment, by first author

First author	Aims and objectives	Research design	Process for producing findings	Data supports interpretation	Appropriate method of analysis	Additional comments
Abeid (2014)	yes	yes	yes	yes	yes	
Abramsky (2011)	yes	yes	yes	yes	yes	
Abramsky (2014)	yes	yes	yes	yes	yes	
Abramsky (2019)	yes	yes	yes	yes	yes	
Abramsky (2020)	yes	yes	yes	yes	yes	
Afiaz (2020)	yes	yes	yes	yes	yes	
Agardh (2012)	yes	yes	yes	yes	yes	
Ager (2018)	yes	yes	yes	yes	yes	
Ahmad (2018)	yes	yes	yes	yes	yes	
Akhter (2016)	yes	yes	yes	yes	yes	
Akhter (2020)	yes	yes	yes	yes	yes	
Akinyemi (2020)	yes	yes	yes	yes	yes	
Aklimunnessa (2007)	yes	yes	yes	yes	yes	
Aktaruzzaman (2020)	yes	yes	yes	yes	yes	
Alio (2011)	yes	yes	yes	yes	yes	
Allen (2012)	yes	yes	yes	yes	yes	
Alleyne-Green (2018)	yes	yes	yes	yes	yes	
Alleyne-Green (2019)	yes	yes	yes	yes	yes	
Aloyce (2020)	yes	yes	yes	yes	yes	

Supplementary material:
Risk factors for violence against women in high-prevalence settings: A mixed methods systematic review and meta-synthesis

First author	Aims and objectives	Research design	Process for producing findings	Data supports interpretation	Appropriate method of analysis	Additional comments
Ambramsky (2016)	yes	yes	yes	yes	yes	
Amegbor (2019a)	yes	yes	yes	yes	yes	
Amegbor (2019b)	yes	yes	yes	yes	yes	
Amowitz (2002)	yes	yes	yes	yes	yes	
Andersson (2007)	yes	yes	yes	yes	yes	
Annan (2010)	yes	yes	yes	yes	yes	
Anwary (2015)	yes	yes	yes	yes	yes	
Anyango (2013)	yes	yes	yes	yes	yes	
Atim (2020)	yes	yes	yes	yes	yes	
Bajracharya (2013)	yes	yes	yes	yes	yes	
Bamiwuye (2014)	yes	yes	yes	yes	yes	
Bartels (2010)	yes	yes	yes	yes	yes	
Bates (2004)	yes	yes	yes	yes	yes	
Behrman (2017)	yes	yes	yes	yes	yes	
Bernards (2013)	yes	yes	yes	yes	yes	
Bhuiya (2003)	yes	yes	yes	yes	yes	
Birbeck (2007)	yes	yes	yes	yes	yes	
Black (2019)	yes	yes	yes	yes	yes	
Bott (2012)	yes	yes	yes	yes	yes	
Camargo (2019)	yes	yes	yes	yes	yes	
Cash (2011)	yes	yes	yes	yes	yes	

Supplementary material:
Risk factors for violence against women in high-prevalence settings: A mixed methods systematic review and meta-synthesis

First author	Aims and objectives	Research design	Process for producing findings	Data supports interpretation	Appropriate method of analysis	Additional comments
Chirwa (2020)	yes	yes	yes	yes	yes	
Chowdury (2018)	yes	yes	yes	yes	yes	
Coll (2020)	yes	yes	yes	yes	yes	
Conroy (2016)	yes	yes	yes	yes	yes	
Cools (2017)	yes	yes	yes	yes	yes	
Crombach (2015)	yes	yes	yes	yes	yes	
Dalal (2009)	yes	yes	yes	yes	yes	
Dalal (2013)	yes	yes	yes	yes	yes	
Das (2015)	yes	yes	yes	yes	yes	
De (2020)	yes	yes	yes	yes	yes	
Decker (2015)	yes	yes	yes	yes	yes	
DfID (2013)	yes	yes	yes	yes	yes	
Dugwen (2013)	yes	yes	yes	yes	yes	
Ehiri (2010)	yes	yes	yes	yes	yes	
Elbert (2013)	yes	yes	yes	yes	yes	
Emusu (2009)	yes	yes	yes	yes	yes	
Epstein (2020)	yes	yes	yes	yes	yes	
Epuitai (2019)	yes	yes	yes	yes	yes	
Erickson (2018)	yes	yes	yes	yes	yes	
Eze (2019)	yes	yes	yes	yes	yes	
Falb (2020)	yes	yes	yes	yes	yes	

Mannell J, *et al. BMJ Global Health* 2022; 7:e007704. doi: 10.1136/bmjgh-2021-007704

Supplementary material:
Risk factors for violence against women in high-prevalence settings: A mixed methods systematic review and meta-synthesis

First author	Aims and objectives	Research design	Process for producing findings	Data supports interpretation	Appropriate method of analysis	Additional comments
Fiji Women's Crisis Centre (2011)	yes	yes	yes	yes	yes	
Fine (2019)	yes	yes	yes	yes	yes	
Fleming (2015)	yes	yes	yes	yes	yes	
Francisco (2010)	yes	yes	yes	yes	yes	
FSM Department of Health and Social Affairs (2014)	yes	yes	yes	yes	yes	
Fulu (2013)	yes	yes	yes	yes	yes	
Garcia-Moreno (2006)	yes	yes	yes	yes	yes	
Gibbs (2018)	yes	yes	yes	yes	yes	
Gibbs (2019)	yes	yes	yes	yes	yes	
Grabe (2015)	yes	yes	yes	yes	yes	
Graham (2010)	yes	yes	yes	yes	yes	
Gubi (2020)	yes	yes	yes	yes	yes	
Hague (2011)	yes	yes	yes	yes	yes	
Hamadani (2020)	yes	yes	yes	yes	yes	
Haque (2020)	yes	yes	yes	yes	yes	
Hasan (2014)	yes	yes	yes	yes	yes	
Heath (2014)	yes	yes	yes	yes	yes	
Hendrickson (2018)	yes	yes	yes	yes	yes	
Holmila (2014)	yes	yes	yes	yes	yes	
Human Rights Watch (2009a)	yes	yes	yes	yes	yes	
Human Rights Watch (2009b)	yes	yes	yes	yes	yes	

Supplementary material:
Risk factors for violence against women in high-prevalence settings: A mixed methods systematic review and meta-synthesis

First author	Aims and objectives	Research design	Process for producing findings	Data supports interpretation	Appropriate method of analysis	Additional comments
Human Rights Watch (2013)	yes	yes	yes	yes	yes	
Instituto Nacional de Estadística Avenida (2016)	yes	yes	yes	yes	yes	
International Rescue Committee (2019)	yes	yes	yes	yes	yes	
Islam (2012)	yes	yes	yes	yes	yes	
Islam (2014)	yes	yes	yes	yes	yes	
Islam (2015)	yes	yes	yes	yes	yes	
Islam (2017)	yes	yes	yes	yes	yes	
Islam (2017)	yes	yes	yes	yes	yes	
Islam (2018)	yes	yes	yes	yes	yes	
Izugbara (2018)	yes	yes	no	no	yes	Unusually high odds ratios and confidence intervals
James-Hawkins (2018)	yes	yes	yes	yes	yes	
Jewkes (2018)	yes	yes	yes	yes	yes	
Jewkes (2019)	yes	yes	yes	yes	yes	
Johnson (2009)	yes	yes	yes	yes	yes	
Kabwama (2019)	yes	yes	yes	yes	yes	
Kadengye (2019)	yes	yes	yes	yes	yes	
Kapiga (2017)	yes	yes	yes	yes	yes	
Karamagi (2006)	yes	yes	yes	yes	yes	
Karim (2007)	yes	yes	yes	yes	yes	
Karim (2012)	yes	yes	yes	yes	yes	

Supplementary material:
Risk factors for violence against women in high-prevalence settings: A mixed methods systematic review and meta-synthesis

First author	Aims and objectives	Research design	Process for producing findings	Data supports interpretation	Appropriate method of analysis	Additional comments
Karim (2016)	yes	yes	yes	yes	yes	
Karim (2018)	yes	yes	yes	yes	yes	
Kaye (2005a)	yes	yes	yes	yes	yes	
Kaye (2005b)	yes	yes	yes	yes	yes	
Kazaura (2016)	yes	yes	yes	yes	yes	
Kelly (2012)	yes	yes	yes	yes	yes	
Kelly (2018)	yes	yes	yes	yes	yes	
Kelly (2019)	yes	yes	yes	yes	yes	
Kenny (2019)	yes	yes	yes	yes	yes	
Khan (2002)	yes	yes	yes	yes	yes	
Kiernan (2016)	yes	yes	yes	yes	yes	
Kinyanda (2016)	yes	yes	yes	yes	yes	
Kiwuwa-Muyingo (2020)	yes	yes	yes	yes	yes	
Klomegah (2008)	yes	yes	yes	yes	yes	
Koenig (2003)	yes	yes	yes	yes	yes	
Kohli (2015)	yes	yes	yes	yes	yes	
Kouyoumdjian (2013)	yes	yes	yes	yes	yes	
Kwagala (2013)	yes	yes	yes	yes	yes	
Kwiringira (2018)	yes	yes	yes	yes	yes	
Kyegombe (2014)	yes	yes	yes	yes	yes	
Laisser (2011)	yes	yes	yes	yes	yes	

Supplementary material:
Risk factors for violence against women in high-prevalence settings: A mixed methods systematic review and meta-synthesis

First author	Aims and objectives	Research design	Process for producing findings	Data supports interpretation	Appropriate method of analysis	Additional comments
Laisser (2014)	yes	yes	yes	yes	yes	
Lary (2004)	yes	yes	yes	yes	yes	
Le Roux (2016)	yes	yes	yes	yes	yes	
Lim (2015)	yes	yes	yes	yes	yes	
Logie (2019)	yes	yes	yes	yes	yes	
Lundberg (2012)	yes	yes	yes	yes	yes	
Lundberg (2015)	yes	yes	yes	yes	yes	
MacLachlan (2009)	yes	yes	yes	yes	yes	
Malama (2019)	yes	yes	yes	yes	yes	
Manji (2020)	yes	yes	yes	yes	yes	
Mannell (2020)	yes	yes	yes	yes	yes	
McCloskey (2005)	yes	yes	yes	yes	yes	
McKelvie (2020)	yes	yes	yes	yes	yes	
Meekers (2013)	yes	yes	yes	yes	yes	
Messersmith (2017)	yes	yes	yes	yes	yes	
Miller (2020)	yes	yes	yes	yes	yes	
Mootz (2017)	yes	yes	yes	yes	yes	
Mootz (2018)	yes	yes	yes	yes	yes	
Mulawa (2018a)	yes	yes	yes	yes	yes	
Mulawa (2018b)	yes	yes	yes	yes	yes	
Mulawa (2018c)	yes	yes	yes	yes	yes	

Mannell J, *et al. BMJ Global Health* 2022; 7:e007704. doi: 10.1136/bmjgh-2021-007704

Supplementary material:
Risk factors for violence against women in high-prevalence settings: A mixed methods systematic review and meta-synthesis

First author	Aims and objectives	Research design	Process for producing findings	Data supports interpretation	Appropriate method of analysis	Additional comments
Mullinax (2013)	yes	yes	yes	yes	yes	
Munro-Kramer (2018)	yes	yes	yes	yes	yes	
Murray (2006)	yes	yes	yes	yes	yes	
Murray (2021)	yes	yes	yes	yes	yes	
Murshid (2016)	yes	yes	yes	yes	yes	
Murshid (2016)	yes	yes	yes	yes	yes	
Murshid (2017)	yes	yes	yes	yes	yes	
Murshid (2017)	yes	yes	yes	yes	yes	
Murshid (2018)	yes	yes	yes	yes	yes	
Namy (2017)	yes	yes	yes	yes	yes	
Nandi (2017)	yes	yes	yes	yes	yes	
Naved (2006)	yes	yes	yes	yes	yes	
Naved (2008)	yes	yes	yes	yes	yes	
Naved (2012)	yes	yes	yes	yes	yes	
Naved (2013)	yes	yes	yes	yes	yes	
Naved (2018)	yes	yes	yes	yes	yes	
Naved (2018)	yes	yes	yes	yes	yes	
Nimi (2020)	yes	yes	yes	yes	yes	
Nyato (2019)	yes	yes	yes	yes	yes	
Odimegwu (2018)	yes	yes	yes	yes	yes	
Ogland (2014)	yes	yes	yes	yes	yes	

Supplementary material:
Risk factors for violence against women in high-prevalence settings: A mixed methods systematic review and meta-synthesis

First author	Aims and objectives	Research design	Process for producing findings	Data supports interpretation	Appropriate method of analysis	Additional comments
Okenwa (2010)	yes	yes	yes	yes	yes	
Osinde (2011)	yes	yes	yes	yes	yes	
Parmar (2012)	yes	yes	yes	yes	yes	
Pengpid (2018)	yes	yes	yes	yes	yes	
Peterman (2011)	yes	yes	yes	yes	yes	
Prabhu (2011)	yes	yes	yes	yes	yes	
Qamar (2020)	yes	yes	yes	yes	yes	
Rahman (2011)	yes	yes	yes	yes	yes	
Rahman (2013)	yes	yes	yes	yes	yes	
Rahman (2014)	yes	yes	yes	yes	yes	
Rapp (2012)	yes	yes	yes	yes	yes	
Rees (2017)	yes	yes	yes	yes	yes	
Rees (2018)	yes	yes	yes	yes	yes	
Rees (2015)	yes	yes	yes	yes	yes	
Reese (2017)	yes	yes	yes	yes	yes	
Rehema (2016)	yes	yes	yes	yes	yes	
Reiss (2019)	yes	yes	yes	yes	yes	
Rezwana (2020)	yes	yes	yes	yes	yes	
Rustad (2016)	yes	yes	yes	yes	yes	
Sabri (2019)	yes	yes	yes	yes	yes	
Saile (2013)	yes	yes	yes	yes	yes	

Mannell J, *et al. BMJ Global Health* 2022; 7:e007704. doi: 10.1136/bmjgh-2021-007704

Supplementary material:
Risk factors for violence against women in high-prevalence settings: A mixed methods systematic review and meta-synthesis

First author	Aims and objectives	Research design	Process for producing findings	Data supports interpretation	Appropriate method of analysis	Additional comments
Sambisa (2010)	yes	yes	yes	yes	yes	
Sambisa (2011)	yes	yes	yes	yes	yes	
Samuels (2015)	yes	yes	yes	yes	yes	
Samuels (2019)	yes	yes	yes	yes	yes	
Sanawar (2019)	yes	yes	yes	yes	yes	
Schuler (2008)	yes	yes	yes	yes	yes	
Schuler (2013)	yes	yes	yes	yes	yes	
Schuler (2017)	yes	yes	yes	yes	yes	
Schuler (2018a)	yes	yes	yes	yes	yes	
Schuler (2018b)	yes	yes	yes	yes	yes	
Schuler (2018)	yes	yes	yes	yes	yes	
Schwitters (2015)	yes	yes	yes	yes	yes	
Scolese (2020)	yes	yes	yes	yes	yes	
Secretariat of the Pacific Community (2010)	yes	yes	yes	yes	yes	
Semenza (2019)	yes	yes	yes	yes	yes	
Shinabarger Reed (2008)	yes	yes	yes	yes	yes	
Shuaib (2012)	yes	yes	yes	yes	yes	
Sichimba (2020)	yes	yes	yes	yes	yes	
Sigalla (2017)	yes	yes	yes	yes	yes	
Sileo (2019)	yes	yes	yes	yes	yes	
Silverman (2007)	yes	yes	yes	yes	yes	

Mannell J, *et al. BMJ Global Health* 2022; 7:e007704. doi: 10.1136/bmjgh-2021-007704

Supplementary material:
Risk factors for violence against women in high-prevalence settings: A mixed methods systematic review and meta-synthesis

First author	Aims and objectives	Research design	Process for producing findings	Data supports interpretation	Appropriate method of analysis	Additional comments
Smith (2019)	yes	yes	yes	yes	yes	
Solomon Islands Ministry of Women Youth and Children's Affairs (2008)	yes	yes	yes	yes	yes	
Speizer (2010)	yes	yes	yes	yes	yes	
Stake (2020)	yes	yes	yes	yes	yes	
Stark (2019)	yes	yes	yes	yes	yes	
Stieglitz (2011)	yes	yes	yes	yes	yes	
Stieglitz (2018)	yes	yes	yes	yes	yes	
Stiglitz (2012)	yes	yes	yes	yes	yes	
Stöckl (2010)	yes	yes	yes	yes	yes	
Stöckl (2014)	yes	yes	yes	yes	yes	
Strønen (2016)	yes	yes	yes	yes	yes	
Tanabe (2015)	yes	yes	yes	yes	yes	
Tiruneh (2018)	yes	yes	yes	yes	yes	
Tlapek (2015)	yes	yes	yes	yes	yes	
Tumwesigye (2012)	yes	yes	yes	yes	yes	
Valentine (2019)	yes	yes	yes	yes	yes	
VanderEnde (2015)	yes	yes	yes	yes	yes	
Vanuatu National Statistics Office (2011)	yes	yes	yes	yes	yes	
Vyas (2014)	yes	yes	yes	yes	yes	
Vyas (2015a)	yes	yes	yes	yes	yes	
Vyas (2015b)	yes	yes	yes	yes	yes	

Mannell J, *et al. BMJ Global Health* 2022; 7:e007704. doi: 10.1136/bmjgh-2021-007704

Supplementary material:
Risk factors for violence against women in high-prevalence settings: A mixed methods systematic review and meta-synthesis

First author	Aims and objectives	Research design	Process for producing findings	Data supports interpretation	Appropriate method of analysis	Additional comments
Vyas (2016)	yes	yes	yes	yes	yes	
Vyas (2018)	yes	yes	yes	yes	yes	
Wadji (2020)	yes	yes	yes	yes	yes	
Wandera (2015)	yes	yes	yes	yes	yes	
Wilson (2019)	yes	yes	yes	yes	yes	
Yaya (2019a)	yes	yes	yes	yes	yes	
Yaya (2019b)	yes	yes	yes	yes	yes	
Young (2018)	yes	yes	yes	yes	yes	
Yount (2016)	yes	yes	yes	yes	yes	
Yount (2018a)	yes	yes	yes	yes	yes	
Yount (2018b)	yes	yes	yes	yes	yes	
Zablotska (2009)	yes	yes	yes	yes	yes	
Zegenhagen (2019)	yes	yes	yes	yes	yes	
